# Phase II randomized, double blind, placebo controlled, clinical trial of safety and immunogenicity of an inactivated SARS-CoV-2 vaccine FAKHRAVAC in adults aged 18–70 years

**DOI:** 10.1186/s12879-023-08079-1

**Published:** 2023-02-24

**Authors:** Fatemeh Gholami, Ramin Hamidi Farahani, Ahmad Karimi Rahjerdi, Mohammadreza Ahi, Ali Sheidaei, Kimiya Gohari, Zahra Rahimi, Akram Ansarifar, Pouria Basiri, Milad Moradi, Arash Jahangiri, Kosar Naderi, Soheil Ghasemi, Pezhman Khatami, Mohsen Honari, Samane Khodaverdloo, Mohammad Shooshtari, Hajar Mehr Azin, Sohrab Moradi, Batool Shafaghi, Hossein Allahyari, Arina Monazah, Ali Khodaei Poor, Zahra Taghva, Hooman Bakhshande, Mohammad Karimi Nia, Masoud Solaymani Dodaran, Mohsen Forooghizade

**Affiliations:** 1 Department of Epidemiology, School of Public Health, Iran University of Medical Sciences, Tehran, Iran; 2 Clinical Trial Center of Iran University of Medical Sciences, Tehran, Iran; 3 AJA University of Medical Sciences, Tehran, Iran; 4 Milad Daro Noor Pharmaceutical (MDNP) Company IR, Tehran, Iran; 5 Stem Cell Technology Research Center (STRC), Tehran, Iran; 6 Malek Ashtar University of Technology, Tehran, Iran

**Keywords:** COVID-19, SARS-CoV-2, Inactivated vaccine, Phase II clinical trial

## Abstract

**Background:**

The FAKHRAVAC®, an inactivated SARS-CoV-2 vaccine, was assessed for safety and immunogenicity in a phase II trial.

**Methods:**

We did a phase II, single-centered, randomized, double-blind, placebo-controlled clinical trial of the FAKHRAVAC inactivated SARS-CoV-2 vaccine on adults aged 18 to 70. The two parallel groups received two intramuscular injections of either a 10-µg vaccine or a placebo at 2-week intervals. The participants' immunogenicity responses and the occurrence of solicited and unsolicited adverse events were compared over the study period of up to 6 months. Immunogenicity outcomes include serum neutralizing antibody activity and specific IgG antibody levels.

**Results:**

Five hundred eligible participants were randomly (1:1) assigned to vaccine or placebo groups. The median age of the participants was 36 years, and 75% were male. The most frequent local adverse reaction was tenderness (21.29% after the first dose and 8.52% after the second dose), and the most frequent systemic adverse reaction was headache (11.24% after the first dose and 8.94% after the second dose). Neutralizing antibody titers two and four weeks after the second injection in the vaccine group showed about 3 and 6 times increase compared to the placebo group (GMR = 2.69, 95% CI 2.32–3.12, N:309) and (GMR = 5.51, 95% CI 3.94–8.35, N:285). A four-fold increase in the neutralizing antibody titer was seen in 69.6% and 73.4% of the participants in the vaccine group two and four weeks after the second dose, respectively. Specific ELIZA antibody response against a combination of S1 and RBD antigens 4 weeks after the second injection increased more than three times in the vaccine compared to the placebo group (GMR = 3.34, 95% CI 2.5–4.47, N:142).

**Conclusions:**

FAKHRAVAC® is safe and induces a significant humoral immune response to the SARS-CoV-2 virus at 10-µg antigen dose in adults aged 18–70. A phase III trial is needed to assess the clinical efficacy.

*Trial registration*: Trial Registry Number: Ref., IRCT20210206050259N2 (http://irct.ir; registered on 08/06/2021)

**Supplementary Information:**

The online version contains supplementary material available at 10.1186/s12879-023-08079-1.

## Introduction

Different vaccine platforms have been used to develop vaccines against SARS-CoV-2 [1–3]. Inactivated vaccines make a major part of WHO's Emergency Use Listing (EUL) [4]. Though they need a great amount of time and facilities for production, they usually do not require advanced technologies and have simple storage and transfer conditions [5–7] and were among the first that became available for use in the COVID-19 pandemic [8].

FAKHRAVAC is an inactivated vaccine that was developed early in the course of the COVID-19 pandemic. The vaccine seed was isolated from Iranian inpatient COVID-19 cases in March 2020 and cultivated using Vero cells. A fast protein liquid chromatography (FPLC) method was used to purify the inactivated viral suspension [9]. Aluminum sulfate was used as the adjuvant. Previous studies have shown that the 10-µg/dose vaccine strengths were safe and provided the optimal immunogenicity in the phase I trial (manuscript under review; Trial Registration ID: IRCT20210206050259N1).

Accumulating safety data is the necessary next step in developing new vaccines [10]. Phase I trial of FAKHRAVAC provided limited safety and immunogenicity data in its first human use. In the current study, we have examined the safety and immunogenicity of FAKHRAVAC compared to a placebo in a larger population aged 18 to 70.

## Methods

### Study design and setting

We did a phase II, single-centered, randomized, double-blind, parallel groups, placebo-controlled clinical trial of FAKHRAVAC inactivated SARS-CoV-2 vaccine on adults aged 18 to 70. The study protocol was approved by the National Ethics Committee for Research in Medical Sciences (approval number IR.NREC.1400.003, June 8, 2021). The trial was registered in the Iranian Registry of Clinical Trials [http://irct.ir; Registration Number: Ref., IRCT20210206050259N2 (registered on 08/06/2021)].

### Participants

Participants were enrolled through a website between May and July 2021. Following declaration of willingness to participate in the trial and an initial online screening, potentially eligible volunteers were invited to attend the trial unit for further psychological, clinical, and laboratory evaluations and to sign a written informed consent. At the psychological assessment session, the participants’ mental capability to give consent was assessed before proceeding with the rest of the trial. Major inclusion criteria were age between 18 and 70 with negative serology (anti-nucleocapsid antibody) and no clinical history for COVID-19, and negative RT-PCR test at the time of recruitment. Major exclusion criteria were pregnancy, breastfeeding, history of allergic diseases and reactions, having any active uncontrolled diseases, malignancy, and close contact with a person with confirmed COVID-19 for a maximum of 2 weeks before the screening day. A detailed description of eligibility criteria has been presented in the Study protocol [Additional file 1: Inclusion and Exclusion (Eligibility) criteria (Pages: 2–5)].

### Randomization and masking

We used permuted block randomization to allocate eligible participants to the vaccine and placebo groups. Block sizes of four were used and each block contained an equal number of alternative interventions. Rand function in excel was used to generate the random sequence within each block. Randomly generated four-digit unique numbers called “randomization codes” were used to conceal the randomization sequence. An independent study epidemiologist created the random sequence and had access to it for emergency unblinding. The identical vaccine and placebo single-injection vials were labeled using the randomization codes. These codes were also embedded in the study software and successively assigned to the eligible participants, once they successfully pass all the stages before receiving the injection and their eligibility for enrollment was confirmed by the research physician. Therefore all participants and the research team were blinded to the study group allocation.

We used a pair of unique codes to label every blood specimen collected for immunogenicity assessment. One of the pairs was put on the paper Case Report Form (CRF) in its relevant field, indicating the type and timing of the immunogenicity test, and the other was on the collection tube. The label used on the collection tube is concealed behind a scratch label and should be revealed by lab offices. The key to the pair was created and kept by the study epidemiologist.

### Procedures

Participants received two intramuscular injections of either a 10-µg vaccine or a placebo (an adjuvant-only preparation) at 2-week intervals. FAKHRAVAC inactivated vaccine was made of SARS-CoV-2 strains isolated from the oropharyngeal Swabs of Iranian hospitalized patients replicated using Vero cells (Cat. # 88020401), a WHO-approved cell line for vaccine production. Aluminum sulfate was used as the adjuvant. The participants' immunogenicity responses and the occurrence of solicited and unsolicited adverse events were assessed over the study period of up to 6 months (see Additional file 1, Diagram of participants' scheduled visits, page 11; Scoring the severity of the adverse reactions, eTable 7 and eTable 8, pages 16–18). Participants reporting any signs and symptoms of suspected COVID-19 disease (see Additional file 1, COVID-19 case definitions, pages 12–13) in their mobile application were actively followed and asked to attend the clinical unit for RT-PCR test. Confirmed cases were excluded from further immunogenicity assessments.

### Outcomes

Our primary safety outcomes were immediate anaphylactic reactions following vaccination and local and systemic adverse events during the week after receiving each vaccine/placebo dose. Participants were kept under close observation in the trial unit for 3 h following vaccination and their vital signs were monitored hourly. Participants were provided with a mobile application to record their daily pre-specified local (pain, tenderness or pain when the injection site is touched, erythema, and swelling or stiffness) and systemic (nausea and vomiting, diarrhea, headache, fatigue, and muscle pain) adverse events up to a week and received daily text reminders to do so. Those who did not complete the relevant forms on the application were contacted and the information was collected by telephone. (see Additional file 1, eTable 5 and eTable 6, pages 14–15).

Our primary and secondary immunogenicity outcomes were serum ELIZA IgG level for SARS-CoV-2 S1-RBD antigen and serum neutralizing activity measured by conventional virus neutralization test two and four weeks after the second injection, respectively. The measurements were repeated three and six months after the first vaccination. Serum lymphokines, including IL6 and other immunologic indices, were assessed in the peripheral blood before and after vaccine/placebo injections. Secondary safety outcomes included laboratory evaluations 1 week after the first (all participants) and second (above 55 year-olds only) injections. Serious Adverse Events (SAEs), Suspected Unexpected Serious Adverse Reaction (SUSARs) and, Medically Attended Adverse Events (MAAEs) were also monitored for up to 6 months [for further details see Additional file 1, Safety Reporting Guidelines (pages: 6–9)].

### Statistical analysis

**Fig. 1:**
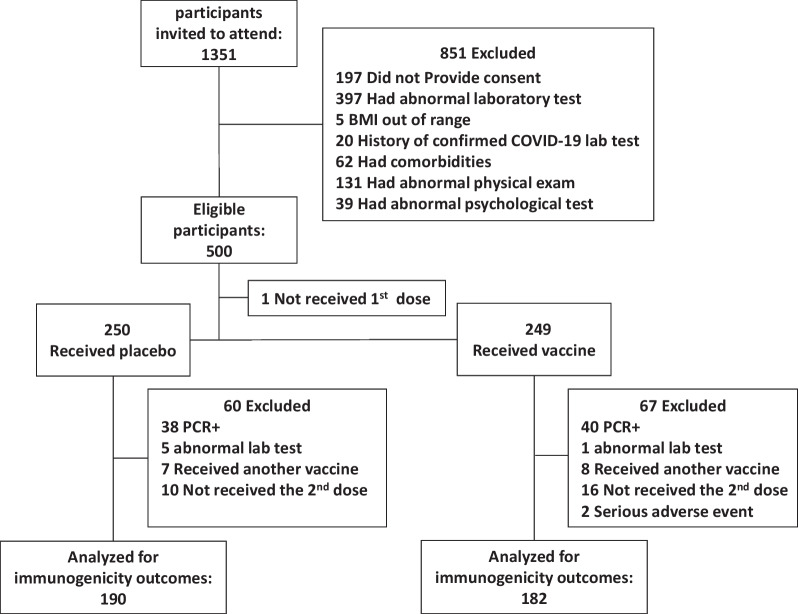
Participant flow diagram of participants

Based on expert opinion, a sample of 500 volunteers was used for phase II (for participants flow diagram and exclusions see Fig. 1). Baseline characteristics were compared to assess their balance in the two randomized groups. All participants who had received a vaccine or a placebo were included in the safety analysis. Per protocol, approach was used to select an efficacy/immunogenicity population. Descriptive statistics were used to report local and systemic reactions. Medians and interquartile range were calculated, reported for quantitative variables, and compared with t-test or Mann–Whitney u-test where appropriate. Categorical variables were reported as numbers and percentages and compared using a chi-squared test. Serum antibody levels were presented as Geometric means and titers, and their 95 percent confidence interval was reported. Geometric mean ratios (GMR) and geometric mean fold ratios (GMFR) and their 95 percent confidence intervals were calculated 4 weeks after the second dose, comparing the vaccine and the placebo groups. Geometric mean fold increase (GMFI) comparing antibody responses at each time point to the baseline was also estimated. STATA version 11 (Stata Corporation, Texas, USA) and R 4.3.2 were used for statistical analysis.

### Role of funding source

The sponsor conducted the immunogenicity tests. The research team coded the serum specimens and concealed their labels before sending them to the lab. The sponsor had no role in data collection, management, analysis, interpretation, and writing of the report.

## Results

Between 21 May and 6 July 2021, 1351 volunteers aged 18–70 were invited and screened for eligibility. Five hundred eligible participants were randomly (1:1) assigned to vaccine or placebo groups (Fig. 1). The median age of the participants was 36 years, and 75% were male. A baseline comparison shows that the two groups have similar characteristics (Table 1).

**Table 1:** Comparison of baseline characteristics

	Vaccine (n = 249)	Placebo (n = 250)	Total
Age, years; n (%)			
18–29	59 (23.6%)	62 (24.8%)	121 (24.2%)
30–39	105 (42%)	112 (44.8%)	217 (43.4%)
40–49	64 (25.6%)	49 (19.6%)	113 (22.6%)
50–59	18 (7.2%)	24 (9.6%)	42 (8.4%)
60–70	4 (1.6%)	3 (1.2%)	7 (1.4%)
Median (q1,q3)	36 (30,42)	35.5 (30,41)	36 (30,42)
Sex; n (%)			
Male	191 (76.4%)	184 (73.6%)	375 (75%)
Female	59 (23.6%)	66 (26.4%)	125 (25%)
Education; n (%)			
Below diploma^a^	11 (4.4%)	12 (4.8%)	23 (4.6%)
Diploma^b^	85 (34%)	100 (40%)	185 (37%)
Bachelor degree	95 (38%)	81 (32.4%)	176 (35.2%)
Master and above	59 (23.6%)	57 (22.8%)	116 (23.2%)
BMI, median (Q1–Q3)	25.75 (23.7–28.4)	25.95 (23.5–28.4)	25.8 (23.55–28.7)
Smoking; n (%)			
Current smoker	65 (26%)	55 (22%)	120 (24%)
Ex-smoker	11 (4.4%)	12 (4.8%)	23 (4.6%)
Never-smoker	174 (69.6%)	183 (73.2%)	357 (71.4%)
Job; n (%)			
Unemployed/Retired	15 (6%)	19 (7.6%)	34 (6.8%)
Government employee	58 (23.2%)	45 (18%)	103 (20.6%)
Private company employee	33 (13.2%)	54 (21.6%)	87 (17.4%)
Self-employed	82 (32.8%)	80 (32%)	162 (32.4%)
Student	23 (9.2%)	22 (8.8%)	45 (9%)
Housewife	39 (15.6%)	30 (12%)	69 (13.8%)

BMI: body mass index

^a^Includes up to 12 years of primary, secondary, and high school

^b^A comprehensive exam held at the end of 12 years of schooling and results in diploma certificate

**Fig. 2:**
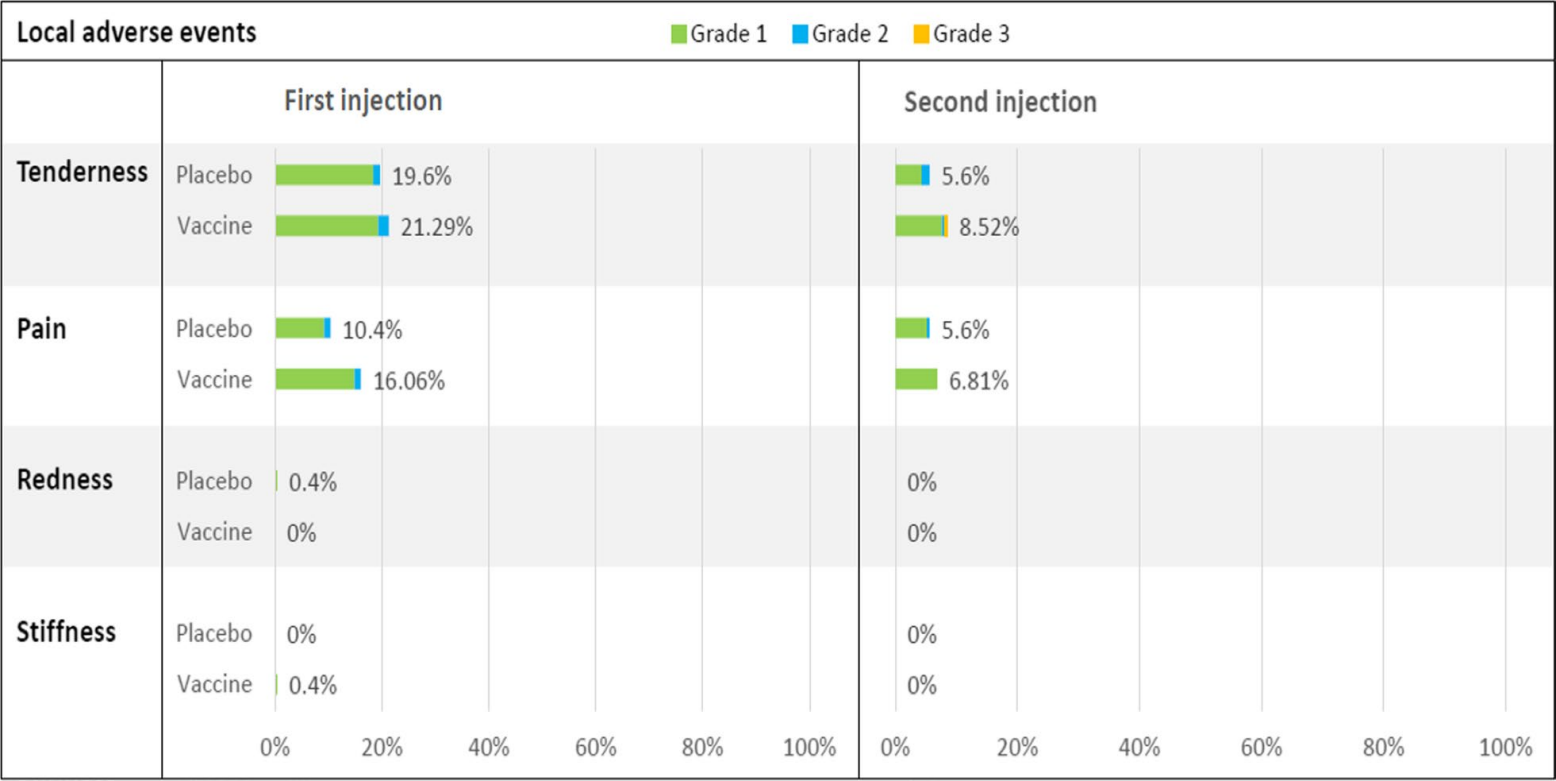
Local reactions within 7 days after each injection

**Fig. 3:**
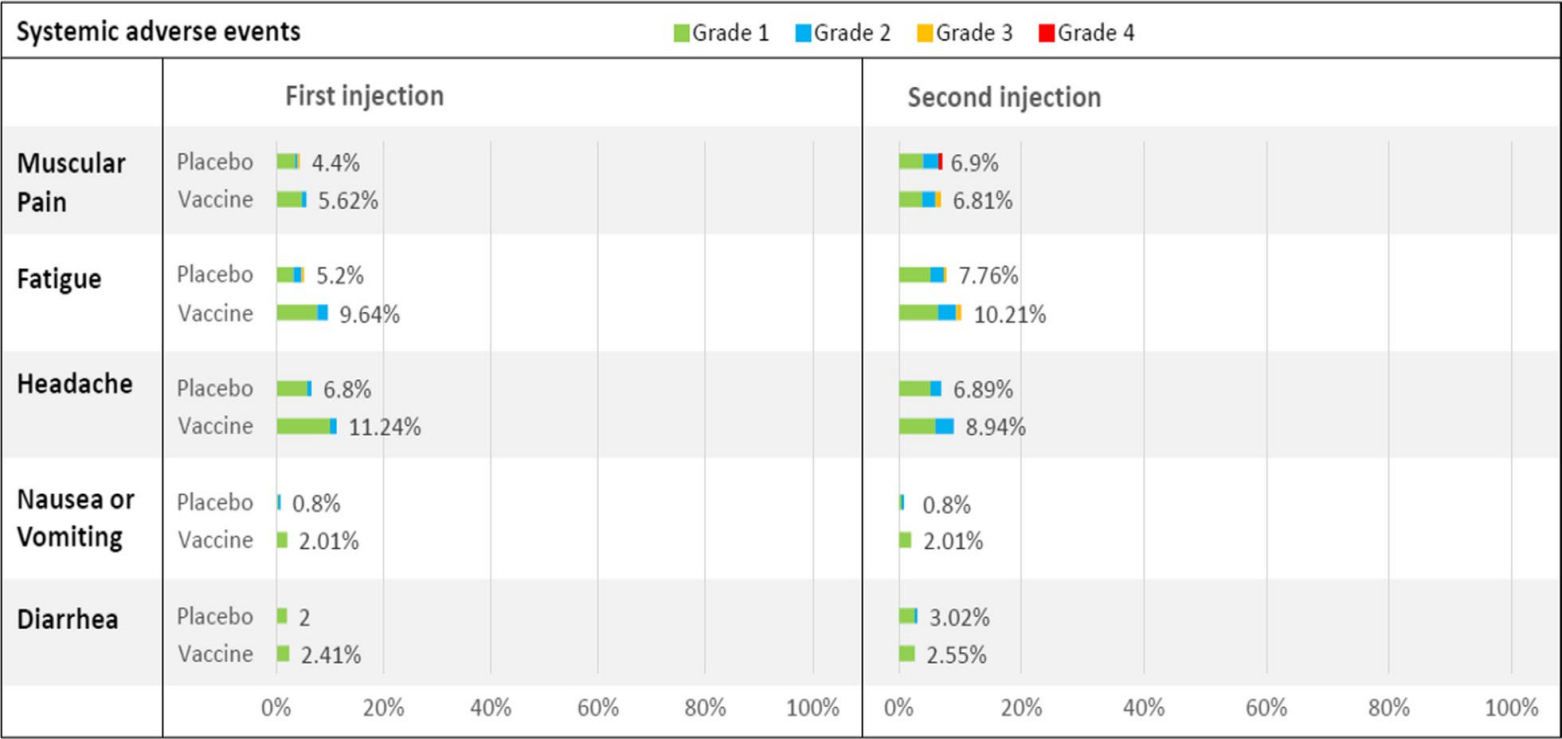
Systemic reactions within 7 days after each injection

The most common local adverse reaction in the first-week post-injection was tenderness (21.29% after the first dose and 8.52% after the second dose) (Fig. 2). The most common systemic adverse reaction was headache (11.24% after the first dose and 8.94% after the second dose), followed by fatigue (9.64% after the first dose and 10.21% after the second dose) (Fig. 3).

Participants reported 128 adverse events resulting in seeking medical attention in 32,063 person-days of follow-up in the vaccine group and 65 in 17,434 in the placebo group. The Incidence Rate Ratio (IRR) of adverse events in the vaccine group compared to the placebo group was 1.07, 95% CI 0.79–1.47. The shorter follow-up period in the placebo group was because the COVID-19 vaccine became available for the general Iranian population around September 2021. We were ethically obliged to inform (unblind) them to leave the study and join the national COVID-19 vaccination program. Five adverse events were categorized as serious. Three were COVID-19 cases, all of which occurred in the placebo group. The other two were hospitalized because of cardiac arrhythmia and nephrolithiasis. The arrhythmia case was observed in a vaccine recipient 5 months after vaccination and regarded as an unrelated event. All adverse events were resolved completely, and non was regarded as definitely related to the vaccine or placebo.

Grade I and II abnormal laboratory findings were observed in 41 vaccine and 85 placebo recipients. Of the 7 grade III abnormalities seen, 4 occurred in the vaccine group and 3 in the placebo group. All the five grade IV abnormalities were reported in the placebo group, two of them in the same person (see Additional file 2, Additional tables and figure. Fig. S6, Table S12). All three participants with grade IV CPK and LDH rise (one person had both abnormalities at the same time) were young men engaged in strenuous exercise (mountain climbing) within the week or two before the injection. Another 22 years old man showed increased level of CRP with no explanation (see Additional file 2: Additional tables and figures. Tables S12–S15). All abnormal laboratory findings were followed up until complete resolution. Overall no obvious trend was observed and the abnormalities were slightly more prevalent in the placebo group. Peripheral blood levels of various cytokines, including IL6 and other immunologic indices, remained relatively unchanged before and after placebo/vaccine injections. (see Additional file 2, Figs. S7–S14).

**Fig. 4:**
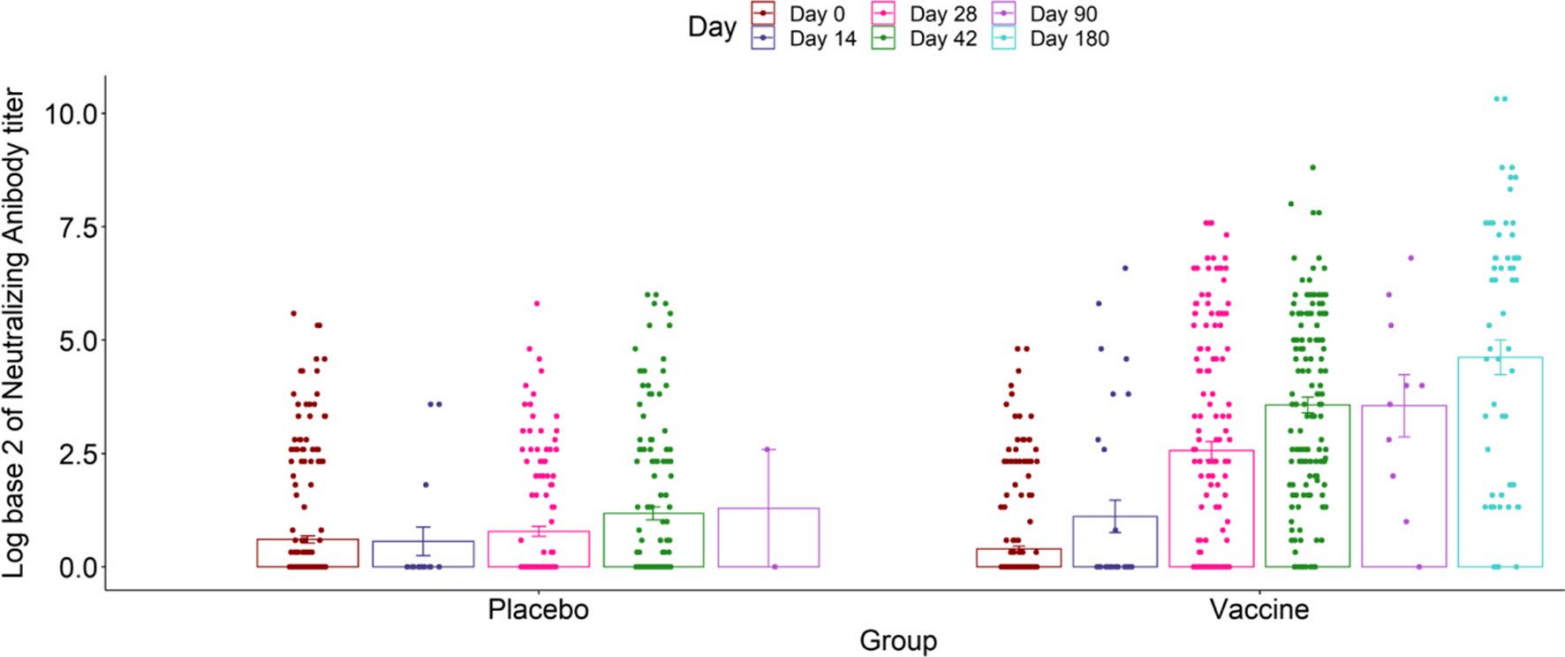
Neutralizing antibodies at different time points among two groups. The dots represent individual participant’s VNT titers in Log base-2 scale. Bars show the means of VNT titers in Log base-2 scale for the group. The error bars represent the confidence interval for the mean

Neutralizing antibody titers two and four weeks after the second injection in the vaccine group showed about 3 and 6 times increase compared to the placebo group (GMR = 2.69, 95% CI 2.32–3.12, N:309) and (GMR = 5.51, 95% CI 3.94–8.35, N:285). Four-fold increase in the neutralizing antibody titer was seen in 69.6% and 73.4% of the participants in the vaccine group two and four weeks after the second dose, respectively (Fig. 4, Table 2). Specific ELIZA antibody response against a combination of S1 and RBD antigens four weeks after the second injection increased more than 3 times in the vaccine compared to the placebo group (GMR = 3.34, 95% CI 2.5–4.47, N:142) (Table 3).

**Table 2:** Neutralizing antibody responses

	Day 0	Day 28	Day 42
GMT (95% CI)			
Placebo	1.52 (1.37–1.70, N:250)	1.72 (1.48–1.99, N:151)	2.26 (1.85–2.75, N:142)
Vaccine	1.31 (1.21–1.43, N:249)	5.90 (4.48–7.78, N:158)	11.88 (9.34–15.10, N:143)
GMR (95% CI)			
Placebo	1	1	1
Vaccine	0.85 (0.44–1.6, N:499) P^1^ = 0.61	2.69 (2.32–3.12, N:309 P^1^ < 0.001	5.51 (3.94–8.35, N:285) P^1^ < 0.001
GMFI (95% CI)			
Placebo	1	1.10 (0.99–1.21, N:151) P^2^ = 0.07	1.49 (0.95–2.64, N:142) P^2^ = 0.26
Vaccine	1	4.62 (3.62–5.90, N:158) P^2^ < 0.001	9.13 (7.68–12.81, N:143) P^2^ < 0.001
GMFR (95% CI)			
Placebo	1	1	1
Vaccine	1	7.54 (9.87–19.91, N:309) P^3^ = 0.01	3.06 (2.26- 5.05, N: 285) P^3^ = 0.004
Seroconversion rate			
Placebo	–	106 (42.4%, 95% CI 36.20–48.79)	23 (16.20%, 95% CI 10.55–23.31)
Vaccine	–	174 (69.6%, 95% CI 63.49–75.24) P^4^ < 0.001	105 (73.42%, 95% CI 65.40–80.46) P^4^ < 0.001

GMT: Geometric Mean Titer; GMR: Geometric Mean Ratio; GMFI: Geometric Mean Fold Increase; GMFR: Geometric Mean Fold Ratio

^1^The P values of testing if the GMR (vaccine group) of each day is equal to 1

^2^The P values of testing if the GMFI of each day and group to its related baseline is equal to 1

^3^The P values of testing if the GMFR (vaccine group) of each day compared with the baseline is equal to 1

^4^The P values of testing if the Seroconversion rate of the vaccine group is statistically different from the placebo on each day

**Table 3:** Antibody responses against S1-RBD antigen

	Day 0	Day 28	Day 42
GM Index (95% CI)			
Placebo	0.42 (0.38–0.47, N:250)	0.47 (0.41–0.53, N:193)	0.68 (0.55–0.84, N:143)
Vaccine	0.38 (0.34–0.42, N:249)	1.08 (0.92–1.27, N:193)	2.28 (1.85–2.8, N:142)
GMR (95% CI)			
Placebo	1	1	1
Vaccine	0.9 (0.78–1.05, N:249) P^1^ = 0.15	2.31 (2.03–2.62, N:193) P^1^ < 0.001	3.34 (2.5–4.47, N:142) P^1^ < 0.001
GMFI (95% CI)			
Placebo	1	1.08 (0.99–1.18, N:193) P = 0.1	1.6 (1.37–1.87, N:143) P < 0.001
Vaccine	1	2.89 (2.55–3.27, N:193) P^2^ < 0.001	5.75 (4.71–7.02, N:142) P^2^ < 0.001
GMFR (95% CI)			
Placebo	1	1	1
Vaccine	1	3.6 (3.06–4.23, N:193) P^3^ < 0.001	2.59 (1.95–3.43, N:142) P^3^ < 0.001

GM: Geometric Mean; GMR: Geometric Mean Ratio; GMFI: Geometric Mean Fold Increase; GMFR: Geometric Mean Fold Ratio

^1^The P values of testing if the GMR (vaccine group) of each day is equal to 1

^2^The P values of testing if the GMFI of each day and group to its related baseline is equal to 1

^3^The P values of testing if the GMFR (vaccine group) of each day compared with the baseline is equal to 1

## Discussion

We found that the 10-µg FAKHRAVAC inactivated SARS-CoV-2 vaccine elicited significant neutralizing antibody response about six times the placebo group (and nine times to the baseline) four weeks after the second dose. Specific antibodies against S and RBD using the ELIZA method also showed a more than three times increase to the placebo and about six times to the baseline. No suspected unexpected serious adverse reaction (SUSAR) was observed in study participants during the follow-up period of up to 6 months. The three hospitalized COVID-19 cases occurred in the placebo group. All abnormal laboratory findings 1 week after each injection were resolved completely.

The follow-up period in our study was 6 months according to the protocol. During this period we collected all medically attended adverse events among study participants and assessed their relationship to the study intervention. However, as COVID-19 vaccines became available for the Iranian general population, it was unethical to deprive the placebo group of the COVID-19 national vaccination programs. Therefore the placebo group in our study were followed for a time period about half the vaccine group.

Local and systemic reactogenicity in the first week after the injections were relatively low compared to other inactivated vaccines [11–14]. FAKHRAVAC showed a satisfactory safety record in laboratory evaluations following vaccine injections, similar to most other vaccines from the same platform [8, 12, 13, 15]. Host cell protein and host cell DNA impurities are the main cause of side effects in the vaccine. We believe our use of the Fast Protein Liquid Chromatography (FPLC) method of vaccine purification [9] resulted in less host-cell protein and DNA in the final product and could partly explain relatively low observed adverse reactions.

We observed a significant neutralizing antibody response in the conventional Virus Neutralization Test (cVNT) two and four weeks after the second dose in the vaccine group compared to the placebo (see Table 2). The decision to assess humoral antibody response 4 weeks after the second injection was made by DSMB during the implementation of the protocol. The reason was that the increase in the specific S1RBD ELIZA antibody response was not still satisfactory 2 weeks after the second injection (2.3 times increase) despite a significant 2.7 times increase in neutralizing antibody response compared to the placebo. The humoral antibody response at 4 weeks continues to rise both in cVNT (5.5 times increase) and S1RBD ELIZA (3.3 times increase) tests compared to the placebo (see Tables 2 and 3). The relatively weaker specific ELIZA antibody responses could be related to the kit used for the assessment.

We showed that FAKHRAVAC elicited a significant immunogenicity response after two doses. Although this does not necessarily translate into the clinical efficacy of the vaccine, there are hints that this may be true. We identified 22 cases of symptomatic PCR-positive COVID-19 disease occurring 2 weeks after the second dose, 8 in the vaccine and 14 in the placebo groups (see Additional file 2: Table S24). Although the results were not statistically significant they provided clues to the protective effect of the vaccine against symptomatic PCR positive COVID-19. We have reported long-term 6-months safety and immunogenicity outcomes in this paper which is one of our strong points compared with other reports of inactivated vaccines [11–13]. Furthermore, we masked the immunology laboratory using pair codes for each blood specimen as described in the method. In this way, we not only protected the identity of the participants for each sample but also prevented establishing any connection between serial samples in each individual over time.

Our study had also some limitations. First, we were unable to continue the follow-up of the placebo group beyond 3 months because approved COVID-19 vaccines became available for the Iranian general population. Second, the number of participants over 50 in our study is relatively low, and the data does not include people over 70, who are an important target population in the vaccination against COVID-19. Third, we did not compare neutralizing antibody titers in our study with that of the convalescent serum samples as positive controls, which could have provided clues to the magnitude of the antibody responses.

In summary, we found that the 10-µg/dose FAKHRAVAC® inactivated SARS-CoV-2 vaccine is safe and induces a significant humoral immune response in adults aged 18–70. A future phase III clinical trial is needed to find out if this observed immunogenicity will translate into clinical efficacy in preventing symptomatic COVID-19 disease.

## Supplementary Information

**Additional file 1.** Inclusion and Exclusion (Eligibility) criteria (pages:2-5). Safety Reporting Guidelines (pages:6-9). Diagram of participants' scheduled visits (page 11). COVID-19 case definitions (pages:12-13). Scoring the severity of local adverse reactions, eTable 5 and Scoring the severity of systemic adverse reactions, eTable 6 (pages:14-15). Scoring the severity of the adverse reactions, eTable 7 and eTable 8 (pages:16-18).

**Additional file 2. Table S12**. Changes in laboratories indices of a 43 years old man receiving the placebo.** Table S13**. Changes in laboratories indices of a 29 years old man receiving the placebo.** Table S14**. Changes in laboratories indices of a 33 years old man receiving the placebo.** Table S15**. Changes in laboratories indices of a 22 years old man receiving the placebo.** Figure S6**. Adverse laboratory events one week after the first injection.** Figure S7-S14**. Peripheral blood flowcytometry for lymphocyte subtypes composition.** Table S24**. Log rank test comparing the occurrence of symptomatic, PCR-positive Covid-19 two weeks after the second injection in study participants receiving 10 µg/dose vaccine with the placebo group.

## Data Availability

Data are available on reasonable request. Individual participant data will be made available when the trial is complete on requests directed to the corresponding author; after the approval of a proposal, data can be shared through a secure online platform.

## References

[1] Wang J, Peng Y, Xu H, Cui Z, Williams RO (2020). The COVID-19 vaccine race: challenges and opportunities in vaccine formulation. AAPS PharmSciTech.

[2] Tregoning JS, Brown E, Cheeseman H, Flight K, Higham S, Lemm N (2020). Vaccines for COVID-19. Clin Exp Immunol.

[3] Koirala A, Joo YJ, Khatami A, Chiu C, Britton PN (2020). Vaccines for COVID-19: the current state of play. Paediatr Respir Rev.

[4] COVID W. vaccine tracker and landscape. 2021.

[5] Pollard AJ, Bijker EM (2021). A guide to vaccinology: from basic principles to new developments. Nat Rev Immunol.

[6] Vetter V, Denizer G, Friedland LR, Krishnan J, Shapiro M (2018). Understanding modern-day vaccines: what you need to know. Ann Med.

[7] Karch CP, Burkhard P (2016). Vaccine technologies: from whole organisms to rationally designed protein assemblies. Biochem Pharmacol.

[8] Xia S, Duan K, Zhang Y, Zhao D, Zhang H, Xie Z (2020). Effect of an inactivated vaccine against SARS-CoV-2 on safety and immunogenicity outcomes: interim analysis of 2 randomized clinical trials. JAMA.

[9] Ghasemi S, Naderi Saffar K, Ebrahimi F, Khatami P, Monazah A, Alizadeh G-A (2021). Development of inactivated FAKHRAVAC® vaccine against SARS-CoV-2 virus: preclinical study in animal models. Vaccines.

[10] Palacios R, Batista AP, Albuquerque CSN, Patiño EG, Santos JdP, Tilli Reis Pessoa Conde M, et al. Efficacy and safety of a COVID-19 inactivated vaccine in healthcare professionals in Brazil: the PROFISCOV study. 2021.

[11] Zhang Y, Zeng G, Pan H, Li C, Hu Y, Chu K (2021). Safety, tolerability, and immunogenicity of an inactivated SARS-CoV-2 vaccine in healthy adults aged 18–59 years: a randomised, double-blind, placebo-controlled, phase 1/2 clinical trial. Lancet Infect Dis.

[12] Xia S, Zhang Y, Wang Y, Wang H, Yang Y, Gao GF (2021). Safety and immunogenicity of an inactivated SARS-CoV-2 vaccine, BBIBP-CorV: a randomised, double-blind, placebo-controlled, phase 1/2 trial. Lancet Infect Dis.

[13] Wu Z, Hu Y, Xu M, Chen Z, Yang W, Jiang Z (2021). Safety, tolerability, and immunogenicity of an inactivated SARS-CoV-2 vaccine (CoronaVac) in healthy adults aged 60 years and older: a randomised, double-blind, placebo-controlled, phase 1/2 clinical trial. Lancet Infect Dis.

[14] Pan H-X, Liu J-K, Huang B-Y, Li G-F, Chang X-Y, Liu Y-F (2021). Immunogenicity and safety of a severe acute respiratory syndrome coronavirus 2 inactivated vaccine in healthy adults: randomized, double-blind, and placebo-controlled phase 1 and phase 2 clinical trials. Chin Med J.

[15] Yun J, Jung YH, Shin SH, Song IG, Lee YA, Shin CH (2021). Impact of very preterm birth and post-discharge growth on cardiometabolic outcomes at school age: a retrospective cohort study. BMC Pediatr.

