# Supplementary material for: Phase II randomized, double blind, placebo controlled, clinical trial of safety and immunogenicity of an inactivated SARS-CoV-2 vaccine FAKHRAVAC in adults aged 18–70 years
Source: BMC Infect Dis. 2023 Feb 24;23:118. doi: 10.1186/s12879-023-08079-1 (PMC9951829; doi:10.1186/s12879-023-08079-1)
Supplement: Supplementary file 1 — Additional file 1. Inclusion and Exclusion (Eligibility) criteria (pages:2-5). Safety Reporting Guidelines (pages:6-9). Diagram of participants' scheduled visits (page 11). COVID-19 case definitions (pages:12-13). Scoring the severity of local adverse reactions, eTable 5 and Scoring the severity of systemic adverse reactions, eTable 6 (pages:14-15). Scoring the severity of the adverse reactions, eTable 7 and eTable 8 (pages:16-18). [file 12879_2023_8079_MOESM1_ESM.pdf]

**Phase II randomized, double blind, placebo controlled, clinical trial of safety and immunogenicity of an inactivated SARS-CoV-2 vaccine FAKHRAVAC in adults aged 18-70 years**

## Supplementary Appendix


| Contents | Page number |
|---|---|
| Inclusion and Exclusion (Eligibility) criteria | **2-5** |
| Safety Reporting Guidelines | **6-8** |
| Superiority by a Margin Tests for the Difference Between Two Proportions | **9-10** |
| Diagram of participants' scheduled visits | **11** |
| COVID-19 case definitions | **12-13** |
| Scoring the severity of local and systemic adverse reactions | **14-15** |
| Scoring the severity of adverse reactions based on laboratory conditions (Serum) | **16-18** |

**Inclusion and Exclusion (Eligibility) criteria**

The inclusion and exclusion criteria are established based on experts' opinions, experiences of phases I and II of human vaccines, and information extracted from the texts.

**Inclusion criteria**

1. Having Iranian citizenship and residence at a distance of 40-50 km from the study place

2. Participants should be able to read and understand informed consent, preferably with a diploma degree or higher

3. Age 18 to 70 years

4. Body mass index between 18 to 35 kg/m2

5. Temperature less than or equal to 37.2 ° C sublingually based on an electronic thermometer

6. Negative IgG and IgM antibody titers against Covid-19 antigen

7. Negative RT-PCR test to detect Covid-19

8. IgG ELISA negative blood test against HIV

9. Heart rate between 60 and 100

10. Systolic blood pressure (between 90 and 140 mm Hg), diastolic blood pressure (between 60 and 90 mm Hg)

11. Signing the informed consent form

12. Accept commitments to reduce the risk of exposure to Covid-19 according to eTable 1.

13. Not pregnant

14. Negative Beta-HCG pregnancy test on the screening day and the vaccination day

15. Using at least one safe method of contraception (condoms, oral contraceptive pills, IUD, Norplant capsule) for women of childbearing (parturition) age 18 to 49 years.

16. Tendency to continue using at least one safe method of contraception (condoms, oral contraceptive pills, intrauterine contraceptive device IUD, Norplant capsule) for women of reproductive age 18 to 49 years to 3 months later from the end of the vaccination period.

17. Participants in the clinical trial should refrain from donating blood or plasma from the time of the first vaccination until three months after the last vaccination.

18. No individual must not participate in another trial during the present study.

19. announcing the person's preparation to stay among the surveyed people in the trial for the whole study period until the research phases are completed within 12 months.

20. Using one of the safest methods of contraception in married men up to 3 months after the last dose of vaccine

eTable 1. List of participants' commitments to reduce the risk of exposure to the Covid 19 virus in the first month after vaccination

| Row | Description of the commitment in the first month after vaccination start |
|---|---|
| 1 | As much as possible, I do not leave the house excluding for essentials issues such as food and medicine and do not have an alternative. |
| 2 | I use leaves or telecommute as much as possible. |
| 3 | If I have to leave the house, I do not use public transport at all, and I use a personal vehicle or a closed taxi (internet) as much as possible. |
| 4 | As much as possible, I avoid attending family parties, excluding very close relatives with whom I have daily contact. |
| 5 | I avoid attending meetings as much as possible. |
| 6 | I will undoubtedly use a mask if I have to leave the house. |
| 7 | I wash my hands regularly with soap and water. |
| 8 | In cases where washing with soap and water is not possible and alcohol is available, I disinfect my hands with 70-degree alcohol. |
| 9 | I will inform the study investigators if my family member or close colleague becomes sick. |

**Exclusion criteria**

1. Those who are medical staff or health care workers involved with Covid-19.

2. Employment as a duty (soldiers) in the subdivisions of the Armed Forces

3. Breastfeeding

4. History of receiving any research vaccine during the 30 days before the screening day

5. History of receiving blood or any blood product or immunoglobulin within three months before the screening day

6. History of Immunodeficiency Disorders (suspected and definite)

7. History of long-term use of immunosuppressive drugs (more than 14 consecutive days) in the last four months leading up to screening day

8. History of long-term use (more than 14 consecutive days) of systemic corticosteroids (equivalent to 10 mg or more of prednisolone daily) or high-dose inhaled steroids (more than 800 µg per day of beclomethasone dipropionate or equivalent) in the last four months leading up to screening day (excluding topical steroids)

9. History of suffering allergic diseases such as angioedema and anaphylactic reactions

10. History of any recognized allergy to the drug or vaccine carriers (Including alum or albumin)

11. History of Recognized allergy to eggs

12. History of autoimmune diseases

13. A history of chemotherapy or radiation therapy in the past five years

14. A history of suffering from cancer in the past five years

15. History of severe psychiatric illnesses

16. History of blood disorders (dyskrasia, coagulopathy, platelet deficiency or disorder, deficiency of blood factors)

17. Suffering from chronic obstructive pulmonary diseases such as asthma and COPD and ischemic cardiovascular disease diagnosed by a specialist

18. Suffering from ischemic cardiovascular disease treated by a physician or having a history of cardiac interventions performed by a cardiac interventionist

19. Having uncontrolled blood pressure (systolic above 140 and diastolic above 90)

20. Having uncontrolled diabetes (HBA1c above six or BS above 140) or being treated with insulin

21. People with congenital anomalies, growth retardation, genetic defects, or severe malnutrition

22. History of chronic neurological diseases in the person or the family (including seizures and epilepsy)

23. People with thyroid disease or a history of thyroid resection, except for controlled hypothyroidism

24. Any history of drug abuse (addiction) or alcohol consumption during the last two years

25. Any disturbances or defects in the results of hematological or biochemical tests performed at the time of screening.

26. History of confirmed Covid-19

27. suffering from the acute febrile disease at the time of vaccination

28. History of allergy to acetaminophen tablets

29. Having acute or chronic hepatitis B and C

30. Pulmonary or extra-pulmonary tuberculosis or receiving anti-tuberculosis treatment

31. Receiving anti-tuberculosis prophylaxis drug

32. History of syncope with blood transfusion or observation of blood

33. A person who has had a splenectomy for any cause or the spleen has a known abnormal function

34. Any close contact with a definitively infected person with Covid-19 for a maximum of two weeks before the day of receiving the first dose of the vaccine

35. Previous history of diseases such as SARS, Mers

36. Those who, for any reason, at the discretion of researchers, do not have the required qualifications to participate in a clinical trial

37. Employment as a duty (soldiers) in the subdivisions of the Armed Forces

## Safety Reporting Guidelines

### Adverse Event (AE)

An adverse event is any unwanted and adverse medical event that occurs to the participants simultaneously participating in this clinical study. In this case, the occurrence of the desired event does not necessarily have a causal relationship with the treatment protocols used in this study.

All adverse events will be recorded and investigated by the safety and data monitoring committee regarding having a causal relationship with the vaccine used in the trial.

The following information will be collected for each adverse event in the CRF.

- Event title/description
- Start date
- End date
- Event severity
- Performed treatments
- The result of the treatments performed
- The importance of the event
- Actions regarding the studied drug.

### Expected Adverse Event

A predictable adverse event is an event whose occurrence could have been predicted based on the information available in vaccine-related documents such as the researcher's brochure. The safety and data monitoring committee will record and review all predictable adverse events for causality with the IMP used in the study.

If these cases cause the participants to withdraw from the study or have a higher than expected prevalence, the researchers will report the issues to the ethics and sponsor committee (Milad Daro Noor Company).

eTable 2. Unexpected Adverse Event report guide

| Complication type | Reporting period | Reporting method |
| --- | --- | --- |
| The duties of the investigator in expected adverse events with higher prevalence than the anticipated level | Immediately after the requirement to withdraw the patient from the trial or the prevalence is higher than the expected level | Announcement of the report by the investigator to the ethics committee and the pharmaceutical company (financial support) |

| | | |
|---|---|---|
| The responsibility of the supporting company in anticipated adverse events with higher prevalence than the expected level | no later than 15 calendar days after the awareness of the pharmaceutical company (financial support) | Announcement of the report by the pharmaceutical company (financial support) to the protocol approving authority (Drug Administration) |

**Serious Adverse Event/Reaction (SAE)**

An event that occurs the following vaccination and leads to death, life-threatening, hospitalization, prolonged hospitalization, permanent or significant disability, or congenital malformation.

eTable 3. Serious Adverse Event Reporting Guide

| Complication type | Reporting period | Reporting method |
|---|---|---|
| The serious adverse event leading to death or life-threatening | Immediate, no later than 24 hours after the investigator becomes aware of the adverse event (via fax, e-mail, etc.) | Announcement of the report by the investigator to the ethics committee and the pharmaceutical company (financial support) |
| The serious adverse event did not lead to death or was not life-threatening to the patient | Immediately, no later than seven calendar days after the investigator becomes aware of the adverse event | Announcement of the report by the investigator to the ethics committee and the pharmaceutical company (financial support) |
| All serious adverse events and follow-up results | The supplementary report contains the relevant information and follow-up results up to 15 calendar days after the pharmaceutical company (financial sponsor) becomes aware of the occurrence of SAE. | Announcement of the report by the pharmaceutical company (financial support) to the protocol approving authority (Drug Administration) |

**Suspected Unexpected Serious Adverse Reaction (SUSAR)**

Serious adverse events are unexpected, and the specifications, characteristics, and severity are not mentioned in the documentation related to the vaccine and the investigator's brochure.

eTable 4. Suspected Unexpected Serious Adverse Reaction SUSAR Guide

| Complication type | Reporting period | Reporting method |
|---|---|---|

| | | |
|---|---|---|
| The serious adverse event leading to death or life-threatening | In the shortest time and at most seven calendar days after the pharmaceutical company (financial sponsor) becomes aware of the occurrence of SUSAR.<br>The supplementary report contains new information, and the follow-ups result up to 15 calendar days after the pharmaceutical company (financial sponsor) is aware of the occurrence of SUSAR. | Announcement of the report by the pharmaceutical company (financial support) to the protocol approving authority (Drug Administration) |
| The serious adverse event did not lead to death or was not life-threatening to the patient | In the shortest possible time and at most 15 calendar days after the pharmaceutical company (financial sponsor) is aware of the occurrence of SUSAR.<br>The supplementary report contains new information and follow-up results in the shortest time after the initial report. | Announcement of the report by the pharmaceutical company (financial support) to the protocol approving authority (Drug Administration) |

If SUSAR leads to death or threatens the patient's life, the sponsor will report the matter to the Ethics Committee and the Food and Drug Administration within the shortest time and within seven days after becoming aware of it by using the relevant form.

If the slightly less severe SUSAR leads to preventive action by the medical team in a life-threatening situation, the maximum reporting time of the sponsor to the Ethics Committee and the Drug office of the Food and Drug Administration can be increased to 15 days.

**Medically Attended Adverse Event (MAAE)**

Some events compel the participants to go to the physician immediately. All adverse events that are classified as severity level 4 have this feature. Refer to (see eTable 5).

If one of these cases is reported by any means (daily/monthly telephone follow-up/volunteer's self-statement during clinical visits), the relevant expert will collect detailed information about this by contacting or directly contacting the participant and the treatment unit. Record in CRF and report to PI as soon as possible.

**Method of dealing with cases that have complications due to receiving IMP**

The study place is Fakhra Clinical Trial Center, and according to the trial place, an ambulance is expected to have the lowest time interval with the hospital's emergency department. However, the following are considered to ensure that immediate threats following vaccination are minimized:

1. An independent room is provided in the inpatient wards to care for trial participants. This clause is included in the contract between Chamran Hospital and the sponsor, and the sponsor bears its costs. In addition, the head of Chamran Hospital is one of the members of the trial steering committee and actively participates in selecting and equipping the trial site.

2. Emergency room at the vaccination site with appropriate facilities to manage possible cases

3. The presence of a special ambulance for the trial in front of the entrance door of the building where the study is conducted

4. Monitoring all participants for at least three hours after receiving IMP and measuring vital signs every hour.

5. Daily non-in-person follow-up of all candidates in the first week after receiving the IMP

6. Monthly non-in-person follow-up of all candidates

7. Considering the on-call physician and providing a mobile phone line to all the candidates

In emergency cases, the individual will be immediately referred to Chamran hospital emergency ward by the physician present at the center or the on-call physician (depending on the time of occurrence of the complication), and the necessary treatment will be done for the person.

**Superiority by a Margin Tests for the Difference Between Two Proportions**

**Numeric Results**

Test Statistic: Z-Test with Unpooled Variance
Hypotheses: H0: P1 - P2 ≤ D0 vs. H1: P1 - P2 > D0

| Power* | N1 | N2 | N | Ref. P2 | P1\|H0 P1.0 | P1\|H1 P1.1 | Sup. Diff D0 | Diff D1 | Alpha |
|---|---|---|---|---|---|---|---|---|---|
| 0.84096 | 151 | 158 | 309 | 0.3 | 0.6 | 0.75 | 0.3 | 0.45 | 0.025 |

* Power was computed using the normal approximation method.

References
Chow, S.C., Shao, J., and Wang, H. 2008. Sample Size Calculations in Clinical Research, Second Edition.
Chapman & Hall/CRC. Boca Raton, Florida.
Farrington, C. P. and Manning, G. 1990. 'Test Statistics and Sample Size Formulae for Comparative Binomial

Trials with Null Hypothesis of Non-Zero Risk Difference or Non-Unity Relative Risk.' Statistics in Medicine, Vol.
9, pages 1447-1454.
Fleiss, J. L., Levin, B., Paik, M.C. 2003. Statistical Methods for Rates and Proportions. Third Edition. John Wiley &
Sons. New York.
Gart, John J. and Nam, Jun-mo. 1988. 'Approximate Interval Estimation of the Ratio in Binomial Parameters: A
Review and Corrections for Skewness.' Biometrics, Volume 44, Issue 2, 323-338.
Gart, John J. and Nam, Jun-mo. 1990. 'Approximate Interval Estimation of the Difference in Binomial Parameters:
Correction for Skewness and Extension to Multiple Tables.' Biometrics, Volume 46, Issue 3, 637-643.
Julious, S. A. and Campbell, M. J. 2012. 'Tutorial in biostatistics: sample sizes for parallel group clinical trials with
binary data.' Statistics in Medicine, 31:2904-2936.
Lachin, John M. 2000. Biostatistical Methods. John Wiley & Sons. New York.
Machin, D., Campbell, M., Fayers, P., and Pinol, A. 1997. Sample Size Tables for Clinical Studies, 2nd Edition.
Blackwell Science. Malden, Mass.
Miettinen, O.S. and Nurminen, M. 1985. 'Comparative analysis of two rates.' Statistics in Medicine 4: 213-226.

Report Definitions
Power is the probability of rejecting a false null hypothesis.
N1 and N2 are the number of items sampled from each population.
N is the total sample size, N1 + N2.
P2 is the proportion for Group 2, which is the standard, reference, or control group.
P1 is the proportion for Group 1, which is the treatment or experimental group. P1.0 is the smallest Group 1
proportion that still yields a superiority conclusion. P1.1 is the proportion for Group 1 under the alternative
hypothesis at which power and sample size calculations are made.
D0 is the superiority difference, P1 - P2, assuming H0. D1 is the difference under the alternative hypothesis used for power and sample size calculations.
Alpha is the probability of rejecting a true null hypothesis.

**Summary Statements**

---

Sample sizes of 151 in Group 1 and 158 in Group 2 achieve 84.096% power to detect a difference of 0.45 when the superiority difference is 0.3. The reference group proportion is 0.3. The treatment group proportion is assumed to be 0.6 under the null hypothesis. The power was computed for the case when the actual treatment group proportion is 0.75. The test statistic used is the one-sided Z test (unpooled). The significance level of the test is 0.025.

eFigure 1. Diagram of participants' scheduled visits

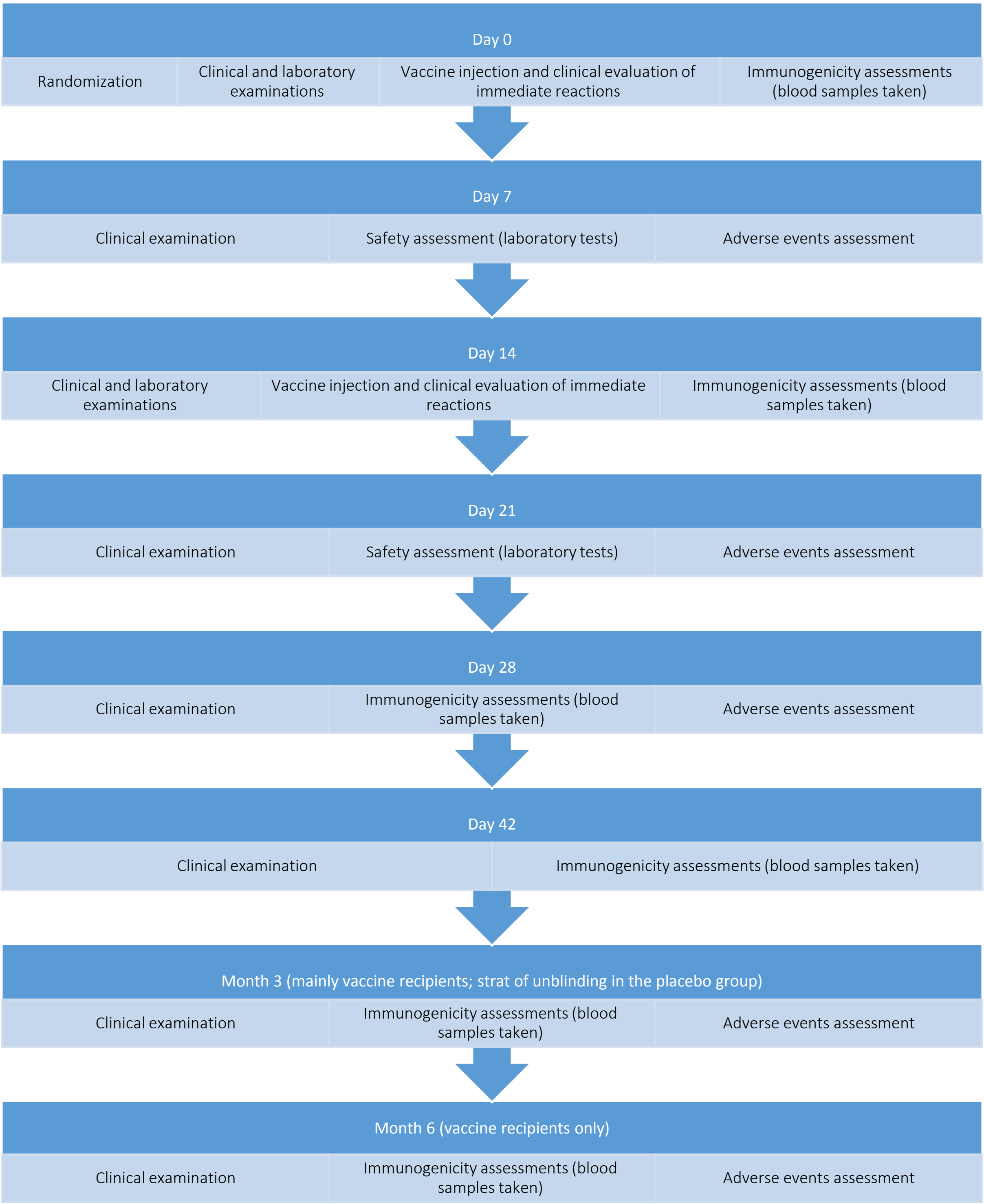

**COVID-19 case definitions**

**Definition of a suspected case**

A) Disease with clinical signs and epidemiological features:

Clinical findings:

Abrupt onset of fever and cough

Or

Sudden onset of at least three or more symptoms such as fever, cough, general weakness/fatigue, headache, myalgia, sore throat, runny nose, dyspnea, anorexia/nausea/vomiting, diarrhea, loss of consciousness

Epidemiological evidence

Accommodation, employment in or travel to areas where the virus is likely to circulate (such as hotels, crowded places, conferences, ceremonies, health centers, etc.) during the last 14 days

B) A patient with severe acute respiratory infection (SARI) needs to be hospitalized with the onset of symptoms within the last 10 days

**Definition of a probable case**

A) A suspected patient who is in close contact with a probable or confirmed patient or a cluster of patients with at least one confirmed case report among them

B) A suspected patient with findings in favor of COVID-19 in medical imaging evaluations such as unilateral or bilateral multilobar infiltrates, particularly peripheral infiltration or ground-glass opacity in CT Scan of the lung or chest radiography (clinically confirmed)

C) A patient experiencing acute loss of sense of smell or taste

D) Death in a patient with suspected COVID-19 (above criteria), not justified by any other reason

Close contact:

A person who has been in contact with a probable or confirmed case within two days before to 14 days after the onset of symptoms in the following circumstances:

1) Face to face contact at a distance of less than 1 meter for at least 15 minutes

2) Direct physical contact with the probable or confirmed case

3) Taking care of probable or confirmed case without using appropriate personal protective equipment

Or

4) In the circumstances other than the above, assessments will be done based on the probability of local transmissions.

**Definition of a confirmed case**

Patient with laboratory confirmation of the presence of SARS-CoV-2, regardless of the appearances of clinical signs and symptoms.

eTable 5. Scoring the severity of local adverse reactions (1)

| Local Reaction to Injectable Product | Mild (Grade 1) | Moderate(Grade 2) | Severe (Grade 3) | Potentially Life Threatening (Grade 4) |
|---|---|---|---|---|
| Pain | Does not interfere with activity | Repeated use of non-narcotic pain reliever > 24 hours or interferes with activity | Any use of narcotic pain reliever or prevents daily activity | Emergency room (ER) visit or hospitalization |
| Tenderness | Mild discomfort to touch | Discomfort with movement | Significant discomfort at rest | ER visit or hospitalization |
| Erythema/Redness * | 2.5 – 5 cm | 5.1 – 10 cm | > 10 cm | Necrosis or exfoliative dermatitis |
| Induration/Swelling ** | 2.5 – 5 cm and does not interfere with activity | 5.1 – 10 cm or interferes with activity | > 10 cm or prevents daily activity | Necrosis |

* In addition to grading the measured local reaction at the greatest single diameter, the measurement should be recorded as a continuous variable. ** Induration/Swelling should be evaluated and graded using the functional scale as well as the actual measurement.

eTable 6. Scoring the severity of systemic adverse reactions (1)

| Systemic (General) | Mild (Grade 1) | Moderate(Grade 2) | Severe (Grade 3) | Potentially Life Threatening (Grade 4) |
|---|---|---|---|---|
| Nausea/vomiting | No interference with activity or 1 – 2 episodes/24 hours | Some interference with activity or > 2 episodes/24 hours | Prevents daily activity, requires outpatient IV hydration | Emergency Room (ER) visit or hospitalization for hypotensive shock |
| Diarrhea | 2 – 3 loose stools or < 400 gms/24 hours | 4 – 5 stools or 400 – 800 gms/24 hours | 6 or more watery stools or > 800 gms/24 hours or requires outpatient IV | ER visit or hospitalization |

| | | | | |
|---|---|---|---|---|
| | | | hydration | |
| Headache | No interference with activity | Repeated use of non-narcotic pain reliever > 24 hours or some interference with activity | Significant; any use of narcotic pain reliever or prevents daily activity | ER visit or hospitalization |
| Fatigue | No interference with activity | Some interference with activity | Significant; prevents daily activity | ER visit or hospitalization |
| Myalgia | No interference with activity | Some interference with activity | Significant; prevents daily activity | ER visit or hospitalization |
| Illness or clinical adverse event (as defined according to applicable regulations) | No interference with activity | Some interference with activity not requiring medical intervention | Prevents daily activity and requires medical intervention | ER visit or hospitalization |

eTable 7. Scoring the severity of adverse reactions based on laboratory conditions (Serum) (1)

| Serum * | Mild (Grade 1) | Moderate (Grade 2) | Severe (Grade 3) | Potentially Life Threatening (Grade 4)** |
|---|---|---|---|---|
| Sodium – Hyponatremia mEq/L | 132 – 134 | 130 – 131 | 125 – 129 | < 125 |
| Sodium – Hypernatremia mEq/L | 144 – 145 | 146 – 147 | 148 – 150 | > 150 |
| Potassium – Hyperkalemia mEq/L | 5.1 – 5.2 | 5.3 – 5.4 | 5.5 – 5.6 | > 5.6 |
| Potassium – Hypokalemia mEq/L | 3.5 – 3.6 | 3.3 – 3.4 | 3.1 – 3.2 | < 3.1 |
| Glucose – Hypoglycemia mg/dL | 65 – 69 | 55 – 64 | 45 – 54 | < 45 |
| Glucose – Hyperglycemia<br>Fasting – mg/dL<br>Random – mg/dL | 100 – 110<br>110 – 125 | 111 – 125<br>126 – 200 | >125<br>>200 | Insulin requirements or hyperosmolar coma |
| Blood Urea Nitrogen<br>BUN mg/dL | 23 – 26 | 27 – 31 | > 31 | Requires dialysis |
| Creatinine – mg/dL | 1.5 – 1.7 | 1.8 – 2.0 | 2.1 – 2.5 | > 2.5 or requires dialysis |
| Calcium – hypocalcemia mg/dL | 8.0 – 8.4 | 7.5 – 7.9 | 7.0 – 7.4 | < 7.0 |
| Calcium – hypercalcemia mg/dL | 10.5 – 11.0 | 11.1 – 11.5 | 11.6 – 12.0 | > 12.0 |
| Magnesium – hypomagnesemia mg/dL | 1.3 – 1.5 | 1.1 – 1.2 | 0.9 – 1.0 | < 0.9 |
| Phosphorous – hypophosphatemia mg/dL | 2.3 – 2.5 | 2.0 – 2.2 | 1.6 – 1.9 | < 1.6 |

| | | | | |
|---|---|---|---|---|
| CPK – mg/dL | 1.25 – 1.5 x ULN*** | 1.6 – 3.0 x ULN | 3.1 –10 x ULN | > 10 x ULN |
| Albumin – Hypoalbuminemia g/dL | 2.8 – 3.1 | 2.5 – 2.7 | < 2.5 | -- |
| Total Protein – Hypoproteinemia g/dL | 5.5 – 6.0 | 5.0 – 5.4 | < 5.0 | -- |
| Alkaline phosphate –<br>increase by factor | 1.1 – 2.0 x ULN | 2.1 – 3.0 x ULN | □3.1 – 10 x ULN | > 10 x ULN |
| Liver Function Tests – ALT, AST<br>increase by factor | 1.1 – 2.5 x ULN | 2.6 – 5.0 x ULN | 5.1 – 10 x ULN | > 10 x ULN |
| Bilirubin – when accompanied<br>by any increase in Liver Function Test<br>increase by factor | 1.1 – 1.25 x ULN | 1.26 – 1.5 x ULN | 1.51 – 1.75 x ULN | > 1.75 x ULN |
| Bilirubin – when Liver Function Test is normal; increase by factor | 1.1 – 1.5 x ULN | 1.6 – 2.0 x ULN | 2.0 – 3.0 x ULN | > 3.0 x ULN |
| Cholesterol | 201 – 210 | 211 – 225 | > 226 | --- |
| Pancreatic enzymes – amylase, lipase | 1.1 – 1.5 x ULN | 1.6 – 2.0 x ULN | 2.1 – 5.0 x ULN | > 5.0 x ULN |

* The laboratory values provided in the tables serve as guidelines and are dependent upon institutional normal parameters. Institutional normal reference ranges should be provided to demonstrate that they are appropriate. ** The clinical signs or symptoms associated with laboratory abnormalities might result in characterization of the laboratory abnormalities as Potentially Life Threatening (Grade 4). For example, a low sodium value that falls within a grade 3 parameter (125-129 mE/L) should be recorded as a grade 4 hyponatremia event if the subject had a new seizure associated with the low sodium value. ***ULN" is the upper limit of the normal range.

eTable 8. Scoring the severity of adverse reactions based on laboratory conditions (Urine) (1)

| Urine * | Mild (Grade 1) | Moderate<br>(Grade 2) | Severe (Grade 3) | Potentially Life Threatening<br>(Grade 4) |
|---|---|---|---|---|
| Protein | Trace | 1+ | 2+ | Hospitalization or |

| | | | | |
|---|---|---|---|---|
| | | | | dialysis |
| Glucose | Trace | 1+ | 2+ | Hospitalization for hyperglycemia |
| Blood (microscopic) – red blood cells per high power field (rbc/hpf) | 1 - 10 | 11 – 50 | > 50 and/or gross blood | Hospitalization or packed red blood cells (PRBC) transfusion |

Reference

1. Food, Administration D. Toxicity grading scale for healthy adult and adolescent volunteers enrolled in preventive vaccine clinical trials. Rockville, MD: Botswana Government Print. 2007.
