# Supplementary material for: Phase II randomized, double blind, placebo controlled, clinical trial of safety and immunogenicity of an inactivated SARS-CoV-2 vaccine FAKHRAVAC in adults aged 18–70 years
Source: BMC Infect Dis. 2023 Feb 24;23:118. doi: 10.1186/s12879-023-08079-1 (PMC9951829; doi:10.1186/s12879-023-08079-1)
Supplement: Supplementary file 2 — Additional file 2. Table S12. Changes in laboratories indices of a 43 years old man receiving the placebo. Table S13. Changes in laboratories indices of a 29 years old man receiving the placebo. Table S14. Changes in laboratories indices of a 33 years old man receiving the placebo. Table S15. Changes in laboratories indices of a 22 years old man receiving the placebo. Figure S6. Adverse laboratory events one week after the first injection. Figure S7-S14. Peripheral blood flowcytometry for lymphocyte subtypes composition. Table S24. Log rank test comparing the occurrence of symptomatic, PCR-positive Covid-19 two weeks after the second injection in study participants receiving 10 µg/dose vaccine with the placebo group. [file 12879_2023_8079_MOESM2_ESM.pdf]

FAKHRAVAC

Phase II

Results

Contents

BASELINE COMPARISONS 7

Participant flow diagram 7

Comparison of participant's baseline characteristics 8

Comparison of baseline vital signs on screening day 8

Comparison of baseline laboratory results on screening day 9

SAFETY OUTCOMES 10

Medically Attended Adverse Events (MAAE) 10

Vital signs at the time of vaccination 10
- First injection 10
- Second injection 11

Local adverse reactions 11
- First injection 11
- Second injection 13

Systemic adverse reactions 14
- First injection 14
- Second injection 15

Laboratory findings 17
- first injection 17
- second injection 21

Immunological indices 23
- Peripheral blood flowcytometry for lymphocyte subtypes composition 23

IMMUNOGENICITY OUTCOMES 27

Neutralization antibody activity 28

Serum ELISA IgG levels for SARS-CoV-2 30
- Serum IgG levels for S1RBD antigen 30
- Serum IgG levels for Nucleocapsid antigen 33

WITHDRAWAL CASES 35

Survival analysis 41

- Log-rank test 41
- Cox proportional hazard regression 42

Index of tables

Table S 1 baseline characteristics of study subjects 8
Table S 2 Comparison of baseline vital signs in vaccine strengths of 2.5 × 10^6 $TCID_{50}$ (10 μg/dose) and placebo 8
Table S 3 Comparison of baseline laboratory results in vaccine strengths of 2.5 × 10^6 $TCID_{50}$ (10 μg/dose) and placebo 9
Table S 4 Frequncy of seeking medical attention or receiving medication in study groups (receiving vaccine strengths of 2.5 × 10^6 $TCID_{50}$ (10 μg/dose) or a placebo) 10
Table S 5 Vital signs at the time of 1st injection in vaccine strengths of 2.5 × 10^6 $TCID_{50}$ (10 μg/dose) and placebo groups 10
Table S 6 Vital signs at the time of 2nd injection in vaccine strengths of 2.5 × 10^6 $TCID_{50}$ (10 μg/dose) and placebo groups 11
Table S 7 Grades of local adverse reactions in the six days following the 1st injection in vaccine strengths of 2.5 × 10^6 $TCID_{50}$ (10 μg/dose) and placebo groups 12
Table S 8 Grades of local adverse reactions in the six days following the 2nd injection in vaccine strengths of 2.5 × 10^6 $TCID_{50}$ (10 μg/dose) and placebo groups 13
Table S 9 Grades of systemic adverse reactions in the six days following the 1st injection in vaccine strengths of 2.5 × 10^6 $TCID_{50}$ (10 μg/dose) and placebo groups 14
Table S 10 Grades of systemic adverse reactions in the six days following the 2nd injection in vaccine strengths of 2.5 × 10^6 $TCID_{50}$ (10 μg/dose) and placebo groups 16
Table S 11 Grading of abnormal laboratory findings one week after the first injection in vaccine strengths of 2.5 × 10^6 TCID50 (10 μg/dose) and placebo groups 17
Table S 12 Changes in laboratories indices of a 43 years old man receiving the placebo 20
Table S 13 Changes in laboratories indices of a 29 years old man receiving the placebo 20
Table S 14 Changes in laboratories indices of a 33 years old man receiving the placebo 20
Table S 15 Changes in laboratories indices of a 22 years old man receiving the placebo 21
Table S 16 Grading of abnormal laboratory findings one week after the second injection in vaccine strengths of 2.5 × 10^6 TCID50 (10 μg/dose) and placebo groups 21
Table S 17 Geometric mean titer, geometric mean ratio (compared to placebo), and geometric mean fold increase (compared to day zero) for serum neutralizing antibody titers and their 95% confidence intervals at predefined time intervals in study groups 27
Table S 18 The proportion of participants with a four-fold increase in neutralizing antibody titer at predefined time intervals in vaccine strengths of 2.5 × 10^6 TCID50 (10 μg/dose) and placebo groups at day 42 28
Table S 19 The proportion of participants with a four-fold increase in neutralizing antibody titer at predefined time intervals in vaccine strengths of 2.5 × 10^6 TCID50 (10 μg/dose) and placebo groups at day 90 28
Table S 20 The proportion of participants with a four-fold increase in neutralizing antibody titer at predefined time intervals in vaccine strengths of 2.5 × 10^6 TCID50 (10 μg/dose) and placebo groups at day 180 28
Table S 21 Geometric mean titer, geometric mean ratio (compared to placebo), and geometric mean fold increase (compared to day zero) for serum specific IgG antibody levels against S1RBD antigen and their 95% confidence intervals at predefined time intervals in study groups 30
Table S 22 Geometric mean, geometric mean ratio (compared to placebo), geometric mean fold increase (compared to day zero), and geometric mean fold ratio for serum specific IgG antibody

LEVELS AGAINST N ANTIGEN AND THEIR 95% CONFIDENCE INTERVALS AT PREDEFINED TIME INTERVALS IN VACCINE STRENGTHS OF 2.5×10^6 $TCID_{50}$ (10 µG/DOSE) AND PLACEBO GROUPS 33
TABLE S 23 DATES AND REASONS FOR WITHDRAWALS IN THE STUDY PARTICIPANTS 35
TABLE S 24 LOG RANK TEST COMPARING THE OCCURRENCE OF SYMPTOMATIC, PCR-POSITIVE COVID-19 TWO WEEKS AFTER THE SECOND INJECTION IN STUDY PARTICIPANTS RECEIVING 10 µG/DOSE VACCINE WITH THE PLACEBO GROUP 41
TABLE S 25 HAZARD RATIO AND ITS 95% CI ESTIMATE DERIVED FROM COX PROPORTIONAL HAZARD REGRESSION MODEL FOR THE OCCURRENCE OF SYMPTOMATIC, PCR-POSITIVE COVID-19 TWO WEEKS AFTER THE SECOND INJECTION IN STUDY PARTICIPANTS RECEIVING 10 µG/DOSE VACCINE COMPARED TO THE PLACEBO GROUP 42

Index of figures

FIGURE S 1 PARTICIPANT FLOW DIAGRAM (REPEAT - HAS BEEN INCLUDED IN THE MAIN MANUSCRIPT) 7
FIGURE S 2 PROPORTION OF PARTICIPANTS EXPERIENCING LOCAL ADVERSE REACTIONS IN THE SIX DAYS FOLLOWING THE 1ST INJECTION IN VACCINE STRENGTHS OF 2.5×10^6 $TCID_{50}$ (10 µG/DOSE) AND PLACEBO GROUPS 11
FIGURE S 3 PROPORTION OF PARTICIPANTS EXPERIENCING LOCAL ADVERSE REACTIONS IN THE SIX DAYS FOLLOWING THE 2ND INJECTION IN VACCINE STRENGTHS OF 2.5×10^6 $TCID_{50}$ (10 µG/DOSE) AND PLACEBO GROUPS 13
FIGURE S 4 PROPORTION OF PARTICIPANTS EXPERIENCING SYSTEMIC ADVERSE REACTIONS IN THE SIX DAYS FOLLOWING THE 1ST INJECTION IN VACCINE STRENGTHS OF 2.5×10^6 $TCID_{50}$ (10 µG/DOSE) AND PLACEBO GROUPS 14
FIGURE S 5 PROPORTION OF PARTICIPANTS EXPERIENCING SYSTEMIC ADVERSE REACTIONS IN THE SIX DAYS FOLLOWING THE 2ND INJECTION IN VACCINE STRENGTHS OF 2.5×10^6 $TCID_{50}$ (10 µG/DOSE) AND PLACEBO GROUPS 15
FIGURE S 6 ADVERSE LABORATORY EVENTS ONE WEEK AFTER THE FIRST INJECTION 19
FIGURE S 7 PROPORTION OF LYMPHOCYTIC POPULATION EXPRESSING CD3 MARKER IN PERIPHERAL BLOOD MEASURED BY FLOWCYTOMETRY AT DAY 0 AND ONE WEEK AFTER FIRST INJECTION IN STUDY GROUPS (A:PLACEBO; B: VACCINE) 23
FIGURE S 8 PROPORTION OF LYMPHOCYTIC POPULATION EXPRESSING CD4 MARKER WITHIN CD3 POSITIVE SUBSET IN PERIPHERAL BLOOD MEASURED BY FLOWCYTOMETRY AT DAY 0 AND ONE WEEK AFTER FIRST INJECTION IN STUDY GROUPS (A:PLACEBO; B: VACCINE) 23
FIGURE S 9 PROPORTION OF LYMPHOCYTIC POPULATION EXPRESSING CD8 MARKER WITHIN CD3 POSITIVE SUBSET IN PERIPHERAL BLOOD MEASURED BY FLOWCYTOMETRY AT DAY 0 AND ONE WEEK AFTER FIRST INJECTION IN STUDY GROUPS (A:PLACEBO; B: VACCINE) 24
FIGURE S 10 CD4/CD8 RATIO WITHIN CD3 POSITIVE LYMPHOCYTIC SUBSET IN PERIPHERAL BLOOD MEASURED BY FLOWCYTOMETRY AT DAY 0 AND ONE WEEK AFTER FIRST INJECTION IN STUDY GROUPS (A:PLACEBO; B: VACCINE) 24
FIGURE S 11 CD4/CD8 RATIO WITHIN LYMPHOCYTIC CELL POPULATION IN PERIPHERAL BLOOD MEASURED BY FLOWCYTOMETRY AT DAY 0 AND ONE WEEK AFTER FIRST INJECTION IN STUDY GROUPS (A:PLACEBO; B: VACCINE) 25
FIGURE S 12 PROPORTION OF LYMPHOCYTIC POPULATION EXPRESSING CD56 MARKER WITHIN CD3 NEGATIVE SUBSET IN PERIPHERAL BLOOD MEASURED BY FLOWCYTOMETRY AT DAY 0 AND ONE WEEK AFTER FIRST INJECTION IN STUDY GROUPS (A:PLACEBO; B: VACCINE) 25
FIGURE S 13 PROPORTION OF CD19 OR CD20 IN PERIPHERAL BLOOD MEASURED BY FLOWCYTOMETRY AT DAY 0 AND ONE WEEK AFTER FIRST INJECTION IN STUDY GROUPS (A:PLACEBO; B: VACCINE) 26

FIGURE S 14 SERUM CONCENTRATIONS PG/ML OF IL-6 IN STUDY PARTICIPANTS AT DAY 0 AND 14 DAYS AFTER 2ND INJECTION IN TWO ADMINISTRATION SCHEDULES AND VACCINE STRENGTH OF 2.5×10^6 $TCID_{50}$ (10 µG/DOSE) AND PLACEBO 26
FIGURE S 15 NEUTRALIZING ANTIBODIES AT DIFFERENT TIME POINTS AMONG TWO GROUPS 29
FIGURE S 16 CHANGES IN SERUM SPECIFIC IGG ANTIBODY LEVELS AGAINST S1RBD ANTIGEN OVER THE STUDY PERIOD FOR EACH INDIVIDUAL PARTICIPANT AND THE GROUP MEAN IN VACCINE STRENGTHS OF 2.5×10^6 $TCID_{50}$ (10 µG/DOSE) AND PLACEBO GROUPS 31
FIGURE S 17 CHANGES IN GROUP MEANS OF SERUM SPECIFIC IGG ANTIBODY LEVELS AGAINST S1RBD ANTIGEN OVER THE STUDY PERIOD IN VACCINE STRENGTHS OF 2.5×10^6 $TCID_{50}$ (10 µG/DOSE) AND PLACEBO GROUPS 31
FIGURE S 18 SERUM SPECIFIC IGG ANTIBODY LEVELS AGAINST S1RBD ANTIGEN OVER THE STUDY PERIOD FOR EACH INDIVIDUAL PARTICIPANT AND THE GROUP MEAN AND ITS 95% CONFIDENCE INTERVAL IN VACCINE STRENGTHS OF 2.5×10^6 $TCID_{50}$ (10 µG/DOSE) AND PLACEBO GROUPS 32
FIGURE S 19 CHANGES IN SERUM SPECIFIC IGG ANTIBODY LEVELS AGAINST N ANTIGEN OVER THE STUDY PERIOD FOR EACH INDIVIDUAL PARTICIPANT AND THE GROUP MEAN IN VACCINE STRENGTHS OF 2.5×10^6 $TCID_{50}$ (10 µG/DOSE) AND PLACEBO GROUPS 33
FIGURE S 20 CHANGES IN GROUP MEANS OF SERUM SPECIFIC IGG ANTIBODY LEVELS AGAINST N ANTIGEN OVER THE STUDY PERIOD IN VACCINE STRENGTHS OF 2.5×10^6 $TCID_{50}$ (10 µG/DOSE) AND PLACEBO GROUPS 34
FIGURE S 21 SERUM SPECIFIC IGG ANTIBODY LEVELS AGAINST N ANTIGEN OVER THE STUDY PERIOD FOR EACH INDIVIDUAL PARTICIPANT AND THE GROUP MEAN AND ITS 95% CONFIDENCE INTERVAL IN VACCINE STRENGTHS OF 2.5×10^6 $TCID_{50}$ (10 µG/DOSE) AND PLACEBO GROUPS 34
FIGURE S 22 KAPLAN-MAYER SURVIVAL CURVE OF THE OCCURRENCE OF SYMPTOMATIC, PCR-POSITIVE COVID-19 TWO WEEKS AFTER THE SECOND INJECTION IN STUDY PARTICIPANTS RECEIVING 10 µG/DOSE VACCINE COMPARED TO THE PLACEBO GROUP 41

Baseline comparisons

Participant flow diagram

Figure S 1 Participant flow diagram (repeat - has been included in the main manuscript)

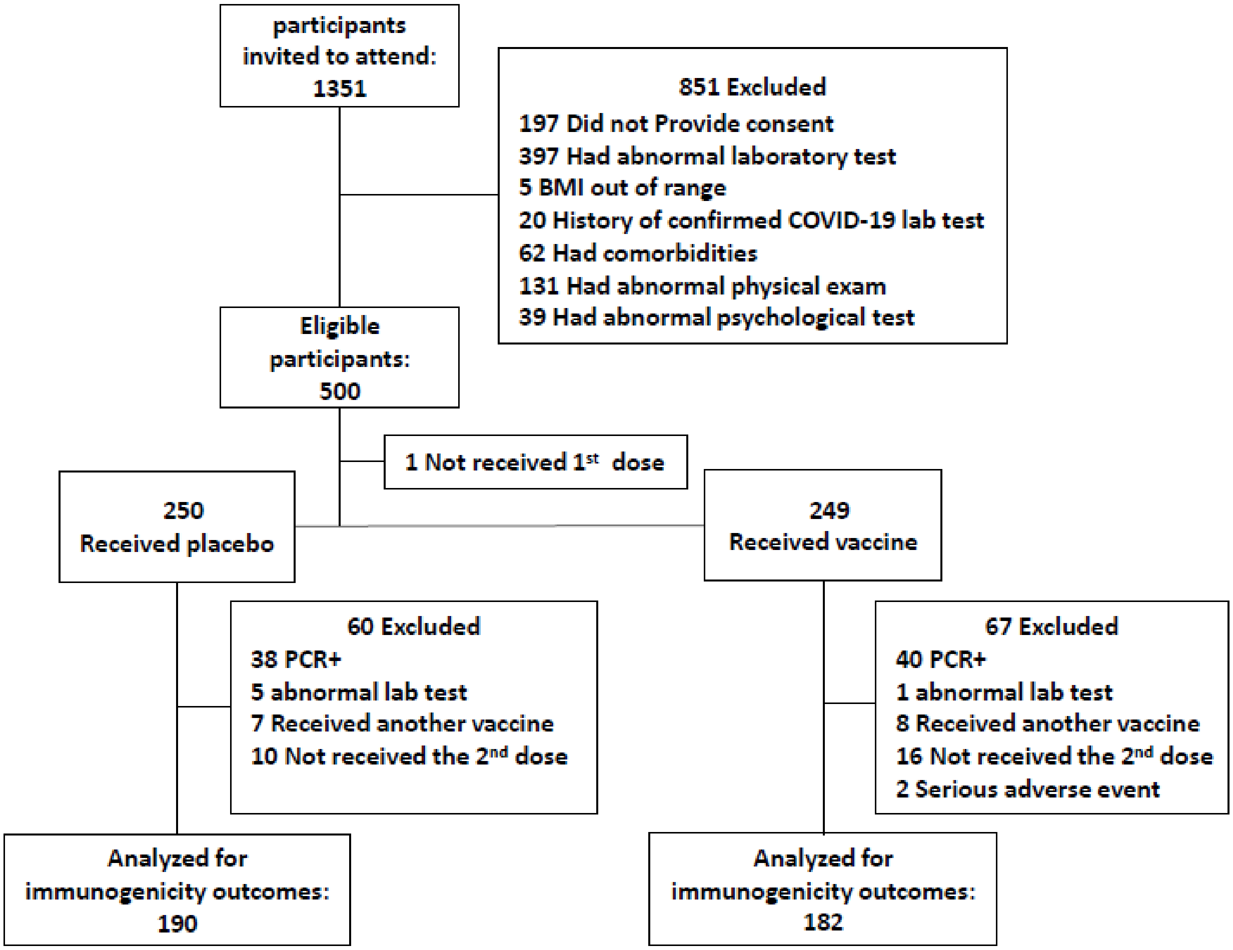

Comparison of participant's baseline characteristics

Table S 1 baseline characteristics of study subjects

Characteristic	Placebo, N = 250[1]	Vaccine, N = 250[1]	N = 500[1]
**Sex**			
Male	184 (74%)	191 (76%)	375 (75%)
Female	66 (26%)	59 (24%)	125 (25%)
**Age (Year)**	36 (30, 41)	36 (30, 42)	36 (30, 42)
**BMI**	25.9 (23.5, 29.1)	25.8 (23.7, 28.4)	25.8 (23.6, 28.7)
**Smoking**			
Current Smoker	35 (14%)	40 (16%)	75 (15%)
Ex-smoker	12 (4.8%)	11 (4.4%)	23 (4.6%)
Never-smoker	183 (73%)	174 (70%)	357 (71%)
Sometimes smoke	20 (8.0%)	25 (10%)	45 (9.0%)
**Education**			
Elementary	12 (4.8%)	11 (4.4%)	23 (4.6%)
Diploma	100 (40%)	85 (34%)	185 (37%)
Bachelor	81 (32%)	95 (38%)	176 (35%)
Master	45 (18%)	53 (21%)	98 (20%)
Doctoral and above	12 (4.8%)	6 (2.4%)	18 (3.6%)
**Job**			
Unemployed/Retired	15 (6.0%)	19 (7.6%)	34 (6.8%)
Government employee	58 (23%)	45 (18%)	103 (21%)
Private employee	33 (13%)	54 (22%)	87 (17%)
Self-employed	82 (33%)	80 (32%)	162 (32%)
Student	23 (9.2%)	22 (8.8%)	45 (9.0%)
Housewife	39 (16%)	30 (12%)	69 (14%)

[1]n (%); Median (Q1, Q3)

Comparison of baseline vital signs on screening day

Table S 2 Comparison of baseline vital signs in vaccine strengths of 2.5 × 10^6 $TCID_{50}$ (10 μg/dose) and placebo

Characteristic	Placebo N = 250[1]	Vaccine, N = 250[1]
**Body Temprature**	36.60 (36.50, 36.70)	36.60 (36.50, 36.70)
**Diastolic Blood Pressure**	80 (72, 85)	79 (72, 84)
**Systolic Blood Pressure**	120 (110, 129)	120 (110, 130)
**Respiratory Rate**	17 (17, 18)	17 (17, 18)
**Heart Rate**	80 (72, 87)	80 (73, 87)
**O2 Saturation**	97 (96, 98)	97 (96, 98)

[1]Median (Q1, Q3)

Comparison of baseline laboratory results on screening day

Table S 3 Comparison of baseline laboratory results in vaccine strengths of 2.5 × 10^6 $TCID_{50}$ (10 µg/dose) and placebo

Characteristic	Placebo N = 250[1]	Vaccine, N = 250[1]
**U/A RBC**		
Normal	244 (98%)	244 (99%)
Grade 1	3 (1.2%)	2 (0.8%)
Grade 2	1 (0.4%)	1 (0.4%)
**AST**		
Normal	250 (100%)	250 (100%)
**ALT**		
Grade 1	4 (1.6%)	1 (0.4%)
Normal	246 (98%)	249 (100%)
**CPK**		
Grade 2	1 (0.4%)	0 (0%)
Normal	249 (100%)	250 (100%)
**ALK**		
Normal	250 (100%)	249 (100%)
**Bilirubin**		
Normal	248 (99%)	249 (100%)
Grade 1	2 (0.8%)	1 (0.4%)
**WBC**		
Normal	248 (99%)	250 (100%)
Increase Grade 1	2 (0.8%)	0 (0%)
**Neutrophils**		
Normal	249 (100%)	248 (100%)
Grade 1	1 (0.4%)	1 (0.4%)
**Platelets**		
Normal	250 (100%)	250 (100%)
**BUN**		
Normal	250 (100%)	250 (100%)
**Potassium**		
Normal	250 (100%)	249 (100%)
**Sodium**		
Normal	250 (100%)	250 (100%)
**Creatinine**		
Normal	249 (100%)	249 (100%)
Grade 1	1 (0.4%)	1 (0.4%)
**ESR**		
Normal	250 (100%)	250 (100%)
**CRP**		
Normal	250 (100%)	249 (100%)
Grade 1	0 (0%)	1 (0.4%)
**LDH**		
Normal	250 (100%)	250 (100%)

[1] n (%)

Safety outcomes

Medically Attended Adverse Events (MAAE)

Table S 4 Frequncy of seeking medical attention or receiving medication in study groups (receiving vaccine strengths of 2.5 × 10^6 TCID$_{50}$ (10 µg/dose) or a placebo)

	Has taken a new medicine for any reason since receiving the vaccine		Has seen a doctor or health center for any reason since receiving the vaccine		Has been hospitalized for any reason since receiving the vaccine	
Characteristic	Placebo N = 250	Vaccine, N = 249	Placebo N = 250	Vaccine, N = 249	Placebo N = 250	Vaccine, N = 249
**Day 1**	2 (0.8%, N: 250)	2 (0.8%, N: 248)	0 (0%, N: 250)	0 (0%, N: 248)	0 (0%, N: 250)	0 (0%, N: 248)
**Day 2**	2 (0.8%, N: 249)	1 (0.4%, N: 248)	2 (0.8%, N: 249)	1 (0.4%, N: 248)	0 (0%, N: 249)	0 (0%, N: 248)
**Day 3**	2 (0.8%, N: 246)	2 (0.8%, N: 247)	2 (0.8%, N: 246)	0 (0%, N: 247)	0 (0%, N: 246)	0 (0%, N: 247)
**Day 4**	1 (0.4%, N: 250)	2 (0.8%, N: 248)	0 (0%, N: 250)	1 (0.4%, N: 248)	0 (0%, N: 250)	0 (0%, N: 248)
**Day 5**	1 (0.4%, N: 250)	0 (0%, N: 249)	0 (0%, N: 250)	0 (0%, N: 249)	0 (0%, N: 250)	0 (0%, N: 249)
**Day 6**	2 (0.8%, N: 249)	0 (0%, N: 248)	0 (0%, N: 249)	0 (0%, N: 248)	0 (0%, N: 249)	0 (0%, N: 248)
**Day 7**	8 (3%, N: 229)	6 (3%, N: 225)	0 (NA%, N: 0)	0 (NA%, N: 0)	0 (NA%, N: 0)	0 (NA%, N: 0)
**Day 14**	6 (2.6%, N: 229)	3 (1.3%, N: 231)	1 (0.4%, N: 229)	1 (0.4%, N: 231)	0 (0%, N: 229)	0 (0%, N: 231)
**Day 15**	0 (0%, N: 217)	1 (0.5%, N: 218)	0 (0%, N: 217)	0 (0%, N: 218)	0 (0%, N: 217)	0 (0%, N: 218)
**Day 16**	0 (0%, N: 219)	1 (0.5%, N: 216)	0 (0%, N: 219)	0 (0%, N: 216)	0 (0%, N: 219)	0 (0%, N: 216)
**Day 17**	2 (0.9%, N: 224)	2 (0.9%, N: 220)	3 (1.3%, N: 224)	1 (0.5%, N: 220)	0 (0%, N: 224)	0 (0%, N: 220)
**Day 18**	0 (0%, N: 221)	3 (1.4%, N: 218)	0 (0%, N: 221)	0 (0%, N: 218)	0 (0%, N: 221)	0 (0%, N: 218)
**Day 19**	2 (0.9%, N: 220)	5 (2.3%, N: 218)	4 (1.8%, N: 220)	1 (0.5%, N: 218)	0 (0%, N: 220)	0 (0%, N: 218)
**Day 20**	2 (0.9%, N: 223)	4 (1.8%, N: 225)	1 (0.4%, N: 223)	4 (1.8%, N: 225)	1 (0.4%, N: 223)	0 (0%, N: 225)
**Day 21**	6 (2.9%, N: 207)	5 (2.5%, N: 204)	1 (0.5%, N: 207)	0 (0%, N: 204)	0 (0%, N: 207)	0 (0%, N: 204)
**Day 28**	7 (4%, N: 191)	7 (4%, N: 192)	0 (NA%, N: 0)	0 (NA%, N: 0)	0 (NA%, N: 0)	0 (NA%, N: 0)
**Month 2**	1 (%, N:)	1(%, N:)	1 (%, N:)	1 (%, N:)	1 (%, N:)	0(%, N:)
**Month 3**	3 (6.5%, N:46)	4 (4.6 %, N:87)	0(0%, N:46)	0 (0%, N:87)	0(0%, N:46)	0(0%, N:87)
**Month 4**	0 (0%, N:0)	6 (5.1%, N: 117)	0(0%, N:0)	6 (5.1%, N:117)	0 (0%, N:0)	0(0%, N:117)
**Month 5**	0 (0%, N:0)	10 (10.6%, N:94)	0(0%, N:0)	17 (18.1%, N9:94)	0 (0%, N:0)	0(0%, N:94)
**Month 6**	0 (0%, N:0)	13 (13.8%, N:94)	0(0%, N:0)	15 (16%, N9:94)	0 (0%, N:0)	2(2.1%, N:94)

Vital signs at the time of vaccination

First injection

Table S 5 Vital signs at the time of 1st injection in vaccine strengths of 2.5 × 10^6 TCID$_{50}$ (10 µg/dose) and placebo groups

	Before IMP		After 1 hour		After 2 hours		After 3 hours	
Characteristic	Placebo N = 250[1]	Vaccine, N = 249[1]	Placebo N = 250[1]	Vaccine, N = 249[1]	Placebo N = 250[1]	Vaccine, N = 249[1]	Placebo N = 250[1]	Vaccine, N = 249[1]
**Body temperature**	36.50 (36.40, 36.70)	36.50 (36.40, 36.60)	36.40 (36.30, 36.50)	36.40 (36.30, 36.50)	36.40 (36.30, 36.50)	36.40 (36.30, 36.50)	36.50 (36.30, 36.60)	36.40 (36.30, 36.60)
**Systolic blood pressure**	120 (111, 129)	121 (110, 129)	117 (108, 127)	117 (108, 125)	119 (110, 128)	118 (110, 125)	120 (110, 128)	120 (112, 129)
**Diastolic blood pressure**	79 (72, 84)	79 (72, 83)	76 (70, 82)	76 (70, 81)	76 (71, 81)	75 (70, 81)	76 (71, 81)	76 (70, 81)
**PO2 saturation**	96 (95, 97)	96 (96, 97)	97 (96, 97)	96 (96, 97)	97 (96, 97)	97 (96, 97)	97 (96, 98)	97 (96, 98)
**Heart rate**	82 (76, 92)	85 (77, 93)	80 (73, 87)	81 (74, 88)	80 (72, 87)	80 (73, 88)	80 (73, 86)	81 (75, 87)
**Respiratory rate**	17 (16, 17)	17 (16, 17)	16 (15, 17)	16 (15, 17)	16 (16, 17)	16 (15, 17)	16 (16, 17)	16 (16, 17)

[1]Median (Q1, Q3)

Second injection

Table S 6 Vital signs at the time of 2nd injection in vaccine strengths of 2.5×10^6 TCID$_{50}$ (10 µg/dose) and placebo groups

	Before IMP		After 1 hour		After 2 hours		After 3 hours	
Characteristic	Placebo N = 250[1]	Vaccine, N = 249[1]	Placebo N = 250[1]	Vaccine, N = 249[1]	Placebo N = 250[1]	Vaccine, N = 249[1]	Placebo N = 250[1]	Vaccine, N = 249[1]
**Body temperature**	36.50 (36.30, 36.60)	36.50 (36.30, 36.60)	36.50 (36.30, 36.60)	36.50 (36.30, 36.60)	36.50 (36.30, 36.70)	36.50 (36.30, 36.60)	36.50 (36.30, 36.60)	36.40 (36.20, 36.60)
**Systolic blood pressure**	115 (104, 123)	115 (106, 124)	115 (105, 124)	112 (105, 121)	116 (106, 125)	117 (109, 124)	117 (109, 128)	120 (112, 129)
**Diastolic blood pressure**	75 (70, 80)	75 (69, 81)	73 (69, 80)	72 (67, 79)	74 (69, 80)	74 (68, 79)	76 (70, 82)	75 (69, 80)
**PO2 saturation**	97 (96, 97)	97 (96, 98)	96 (96, 97)	96 (96, 97)	97 (96, 97)	96 (96, 97)	96 (96, 97)	96 (96, 97)
**Heart rate**	83 (74, 89)	81 (75, 90)	82 (73, 89)	80 (75, 88)	81 (73, 89)	81 (72, 89)	81 (74, 91)	78 (71, 87)
**Respiratory rate**	15 (14, 16)	15 (13, 16)	15 (14, 16)	15 (14, 16)	15 (14, 16)	14 (14, 15)	15 (14, 16)	15 (14, 16)

[1]Median (Q1, Q3)

Local adverse reactions

First injection

Figure S 2 Proportion of participants experiencing local adverse reactions in the six days following the 1st injection in vaccine strengths of 2.5×10^6 TCID$_{50}$ (10 µg/dose) and placebo groups

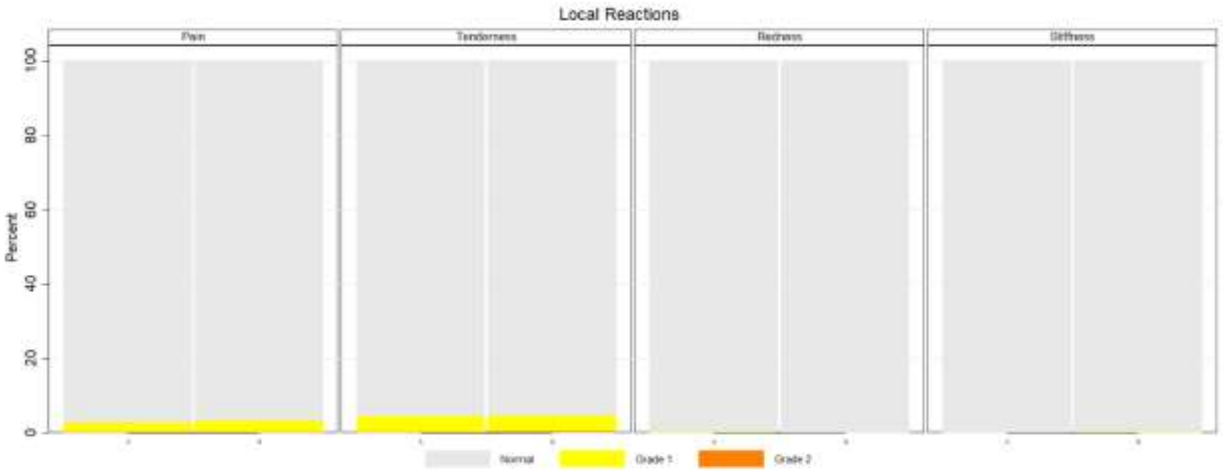

Table S 7 Grades of local adverse reactions in the six days following the 1st injection in vaccine strengths of 2.5 × 10^6 $TCID_{50}$ (10 µg/dose) and placebo groups

	Pain		Tenderness		Redness		Stiffness	
Characteristic	Placebo N = 250	Vaccine, N = 249	Placebo N = 250	Vaccine, N = 249	Placebo, N = 250	Vaccine, N = 249	Placebo, N = 250	Vaccine, N = 249
**Day 1**								
Normal	229 (92%, N: 248)	214 (87%, N: 245)	210 (85%, N: 248)	208 (85%, N: 245)	250 (100%, N: 250)	248 (100%, N: 248)	250 (100%, N: 250)	247 (100%, N: 248)
Grade 1	17 (6.9%, N: 248)	28 (11%, N: 245)	35 (14%, N: 248)	33 (13%, N: 245)	0 (0%, N: 250)	0 (0%, N: 248)	0 (0%, N: 250)	1 (0.4%, N: 248)
Grade 2	2 (0.8%, N: 248)	3 (1.2%, N: 245)	3 (1.2%, N: 248)	4 (1.6%, N: 245)	0 (0%, N: 250)	0 (0%, N: 248)	0 (0%, N: 250)	0 (0%, N: 248)
**Day 2**								
Normal	238 (98%, N: 244)	233 (96%, N: 242)	233 (95%, N: 244)	222 (92%, N: 242)	248 (100%, N: 249)	248 (100%, N: 248)	249 (100%, N: 249)	248 (100%, N: 248)
Grade 1	6 (2.5%, N: 244)	9 (3.7%, N: 242)	11 (4.5%, N: 244)	19 (7.9%, N: 242)	1 (0.4%, N: 249)	0 (0%, N: 248)	0 (0%, N: 249)	0 (0%, N: 248)
Grade 2	0 (0%, N: 244)	0 (0%, N: 242)	0 (0%, N: 244)	1 (0.4%, N: 242)	0 (0%, N: 249)	0 (0%, N: 248)	0 (0%, N: 249)	0 (0%, N: 248)
**Day 3**								
Normal	238 (98%, N: 243)	239 (98%, N: 243)	238 (98%, N: 243)	240 (99%, N: 243)	246 (100%, N: 246)	247 (100%, N: 247)	246 (100%, N: 246)	247 (100%, N: 247)
Grade 1	5 (2.1%, N: 243)	4 (1.6%, N: 243)	5 (2.1%, N: 243)	3 (1.2%, N: 243)	0 (0%, N: 246)	0 (0%, N: 247)	0 (0%, N: 246)	0 (0%, N: 247)
**Day 4**								
Normal	237 (98%, N: 242)	243 (99%, N: 245)	237 (98%, N: 242)	242 (99%, N: 245)	250 (100%, N: 250)	248 (100%, N: 248)	250 (100%, N: 250)	248 (100%, N: 248)
Grade 1	5 (2.1%, N: 242)	2 (0.8%, N: 245)	5 (2.1%, N: 242)	3 (1.2%, N: 245)	0 (0%, N: 250)	0 (0%, N: 248)	0 (0%, N: 250)	0 (0%, N: 248)
**Day 5**								
Normal	246 (100%, N: 247)	245 (100%, N: 246)	243 (98%, N: 247)	243 (99%, N: 246)	250 (100%, N: 250)	249 (100%, N: 249)	250 (100%, N: 250)	249 (100%, N: 249)
Grade 1	1 (0.4%, N: 247)	1 (0.4%, N: 246)	4 (1.6%, N: 247)	3 (1.2%, N: 246)	0 (0%, N: 250)	0 (0%, N: 249)	0 (0%, N: 250)	0 (0%, N: 249)
**Day 6**								
Normal	244 (99%, N: 246)	247 (100%, N: 247)	244 (99%, N: 246)	246 (100%, N: 247)	249 (100%, N: 249)	248 (100%, N: 248)	249 (100%, N: 249)	248 (100%, N: 248)
Grade 1	1 (0.4%, N: 246)	0 (0%, N: 247)	2 (0.8%, N: 246)	1 (0.4%, N: 247)	0 (0%, N: 249)	0 (0%, N: 248)	0 (0%, N: 249)	0 (0%, N: 248)
Grade 2	1 (0.4%, N: 246)	0 (0%, N: 247)	0 (0%, N: 246)	0 (0%, N: 247)	0 (0%, N: 249)	0 (0%, N: 248)	0 (0%, N: 249)	0 (0%, N: 248)

Second injection

Figure S 3 Proportion of participants experiencing local adverse reactions in the six days following the 2nd injection in vaccine strengths of 2.5 × 10^6 $TCID_{50}$ (10 µg/dose) and placebo groups

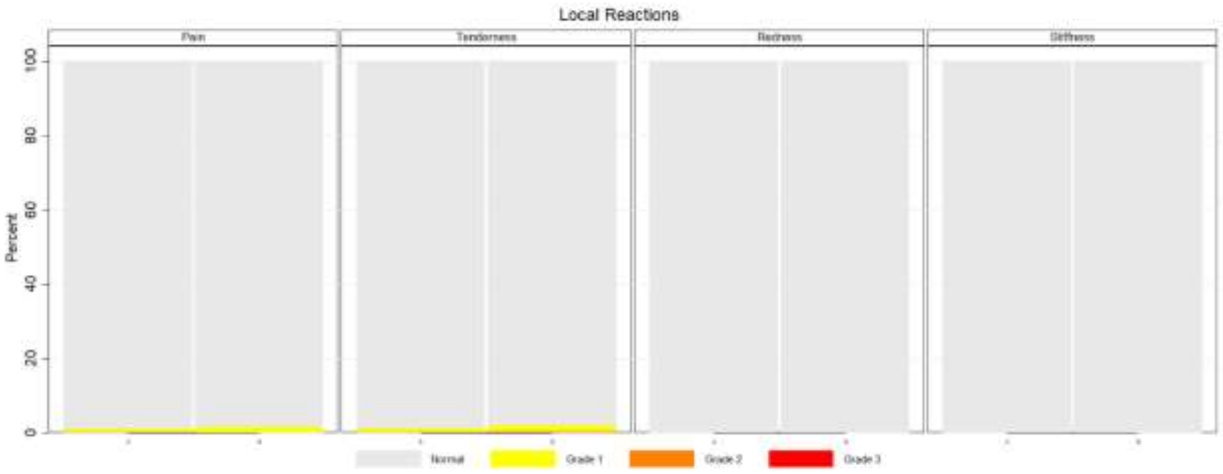

Table S 8 Grades of local adverse reactions in the six days following the 2nd injection in vaccine strengths of 2.5 × 10^6 $TCID_{50}$ (10 µg/dose) and placebo groups

	Pain		Tenderness		Redness		Stiffness	
Characteristic	Placebo, N = 250	Vaccine, N = 249	Placebo, N = 250	Vaccine, N = 249	Placebo, N = 250	Vaccine, N = 249	Placebo, N = 250	Vaccine, N = 249
**Day 1**								
Normal	201 (96%, N: 210)	198 (96%, N: 207)	202 (96%, N: 210)	197 (95%, N: 207)	217 (100%, N: 217)	218 (100%, N: 218)	217 (100%, N: 217)	218 (100%, N: 218)
Grade 1	8 (3.8%, N: 210)	9 (4.3%, N: 207)	5 (2.4%, N: 210)	9 (4.3%, N: 207)	0 (0%, N: 217)	0 (0%, N: 218)	0 (0%, N: 217)	0 (0%, N: 218)
Grade 2	1 (0.5%, N: 210)	0 (0%, N: 207)	3 (1.4%, N: 210)	1 (0.5%, N: 207)	0 (0%, N: 217)	0 (0%, N: 218)	0 (0%, N: 217)	0 (0%, N: 218)
**Day 2**								
Normal	215 (99%, N: 218)	207 (97%, N: 214)	212 (97%, N: 218)	204 (95%, N: 214)	219 (100%, N: 219)	216 (100%, N: 216)	219 (100%, N: 219)	216 (100%, N: 216)
Grade 1	3 (1.4%, N: 218)	7 (3.3%, N: 214)	6 (2.8%, N: 218)	9 (4.2%, N: 214)	0 (0%, N: 219)	0 (0%, N: 216)	0 (0%, N: 219)	0 (0%, N: 216)
Grade 3	0 (0%, N: 218)	0 (0%, N: 214)	0 (0%, N: 218)	1 (0.5%, N: 214)	0 (0%, N: 219)	0 (0%, N: 216)	0 (0%, N: 219)	0 (0%, N: 216)
**Day 3**								
Normal	218 (100%, N: 218)	213 (100%, N: 214)	218 (100%, N: 218)	212 (99%, N: 214)	224 (100%, N: 224)	219 (100%, N: 219)	224 (100%, N: 224)	219 (100%, N: 219)
Grade 1	0 (0%, N: 218)	1 (0.5%, N: 214)	0 (0%, N: 218)	2 (0.9%, N: 214)	0 (0%, N: 224)	0 (0%, N: 219)	0 (0%, N: 224)	0 (0%, N: 219)
**Day 4**								
Normal	217 (100%, N: 218)	212 (100%, N: 213)	218 (100%, N: 218)	211 (99%, N: 213)	221 (100%, N: 221)	218 (100%, N: 218)	221 (100%, N: 221)	218 (100%, N: 218)
Grade 1	1 (0.5%, N: 218)	1 (0.5%, N: 213)	0 (0%, N: 218)	2 (0.9%, N: 213)	0 (0%, N: 221)	0 (0%, N: 218)	0 (0%, N: 221)	0 (0%, N: 218)
**Day 5**								
Normal	219 (100%, N: 219)	213 (100%, N: 213)	219 (100%, N: 219)	213 (100%, N: 213)	220 (100%, N: 220)	217 (100%, N: 217)	220 (100%, N: 220)	217 (100%, N: 217)
**Day 6**								

	Pain		Tenderness		Redness		Stiffness	
Characteristic	Placebo, N = 250	Vaccine, N = 249	Placebo, N = 250	Vaccine, N = 249	Placebo, N = 250	Vaccine, N = 249	Placebo, N = 250	Vaccine, N = 249
Normal	244 (99%, N: 246)	247 (100%, N: 247)	244 (99%, N: 246)	246 (100%, N: 247)	249 (100%, N: 249)	248 (100%, N: 248)	249 (100%, N: 249)	248 (100%, N: 248)
Grade 1	1 (0.4%, N: 246)	0 (0%, N: 247)	2 (0.8%, N: 246)	1 (0.4%, N: 247)	0 (0%, N: 249)	0 (0%, N: 248)	0 (0%, N: 249)	0 (0%, N: 248)
Grade 2	1 (0.4%, N: 246)	0 (0%, N: 247)	0 (0%, N: 246)	0 (0%, N: 247)	0 (0%, N: 249)	0 (0%, N: 248)	0 (0%, N: 249)	0 (0%, N: 248)

Systemic adverse reactions

First injection

Figure S 4 Proportion of participants experiencing systemic adverse reactions in the six days following the 1st injection in vaccine strengths of 2.5 × 10^6 $TCID_{50}$ (10 µg/dose) and placebo groups

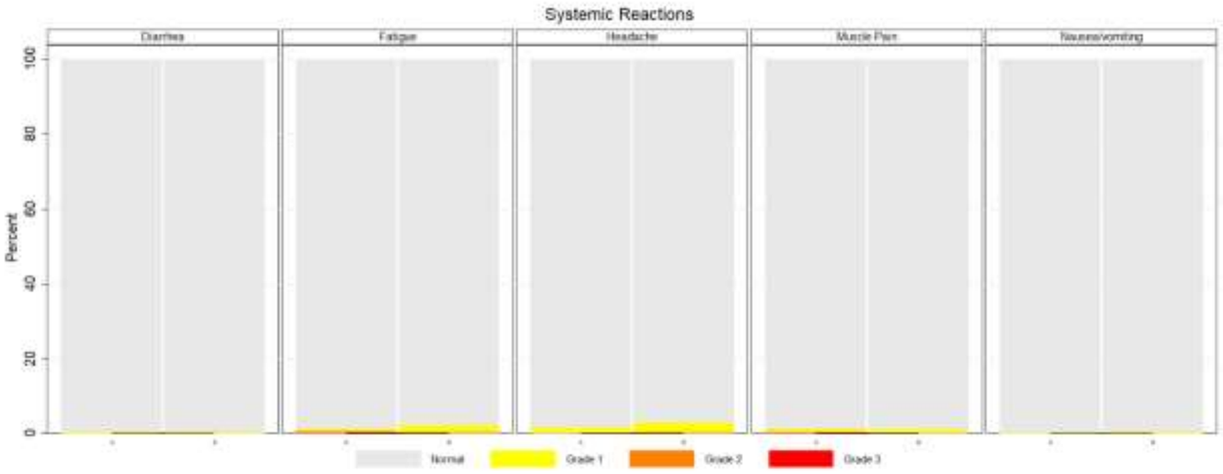

Table S 9 Grades of systemic adverse reactions in the six days following the 1st injection in vaccine strengths of 2.5 × 10^6 $TCID_{50}$ (10 µg/dose) and placebo groups

	Nausea / vomiting		Diarrhea		Headache	
Characteristic	Placebo, N = 250	Vaccine, N = 249	Placebo, N = 250	Vaccine, N = 249	Placebo, N = 250	Vaccine, N = 249
**Day 1**						
Normal	248 (100%, N: 248)	243 (99%, N: 245)	247 (100%, N: 248)	244 (100%, N: 245)	241 (97%, N: 248)	233 (95%, N: 245)
Grade 1	0 (0%, N: 248)	2 (0.8%, N: 245)	1 (0.4%, N: 248)	1 (0.4%, N: 245)	6 (2.4%, N: 248)	10 (4.1%, N: 245)
Grade 2	0 (0%, N: 248)	0 (0%, N: 245)	0 (0%, N: 248)	0 (0%, N: 245)	1 (0.4%, N: 248)	2 (0.8%, N: 245)
**Day 2**						
Normal	244 (100%, N: 244)	241 (100%, N: 242)	243 (100%, N: 244)	239 (99%, N: 242)	240 (98%, N: 244)	238 (98%, N: 242)
Grade 1	0 (0%, N: 244)	1 (0.4%, N: 242)	1 (0.4%, N: 244)	3 (1.2%, N: 242)	4 (1.6%, N: 244)	4 (1.7%, N: 242)
**Day 3**						
Normal	240 (99%, N: 242)	243 (100%, N: 243)	241 (100%, N: 242)	243 (100%, N: 243)	241 (100%, N: 242)	236 (97%, N: 243)
Grade 1	1 (0.4%, N: 242)	0 (0%, N: 243)	1 (0.4%, N: 242)	0 (0%, N: 243)	1 (0.4%, N: 242)	7 (2.9%, N: 243)
Grade 2	1 (0.4%, N: 242)	0 (0%, N: 243)	0 (0%, N: 242)	0 (0%, N: 243)	0 (0%, N: 242)	0 (0%, N: 243)

	Nausea / vomiting		Diarrhea		Headache	
Characteristic	Placebo, N = 250	Vaccine, N = 249	Placebo, N = 250	Vaccine, N = 249	Placebo, N = 250	Vaccine, N = 249
**Day 4**						
Normal	241 (100%, N: 242)	245 (100%, N: 245)	241 (100%, N: 242)	244 (100%, N: 245)	239 (99%, N: 242)	239 (98%, N: 245)
Grade 1	1 (0.4%, N: 242)	0 (0%, N: 245)	1 (0.4%, N: 242)	1 (0.4%, N: 245)	3 (1.2%, N: 242)	6 (2.4%, N: 245)
**Day 5**						
Normal	247 (100%, N: 247)	244 (99%, N: 246)	246 (100%, N: 247)	245 (100%, N: 246)	245 (99%, N: 247)	238 (97%, N: 246)
Grade 1	0 (0%, N: 247)	2 (0.8%, N: 246)	1 (0.4%, N: 247)	1 (0.4%, N: 246)	2 (0.8%, N: 247)	6 (2.4%, N: 246)
Grade 2	0 (0%, N: 247)	0 (0%, N: 246)	0 (0%, N: 247)	0 (0%, N: 246)	0 (0%, N: 247)	2 (0.8%, N: 246)
**Day 6**						
Normal	246 (100%, N: 246)	246 (100%, N: 247)	245 (100%, N: 246)	246 (100%, N: 247)	242 (98%, N: 246)	243 (98%, N: 247)
Grade 1	0 (0%, N: 246)	1 (0.4%, N: 247)	1 (0.4%, N: 246)	1 (0.4%, N: 247)	3 (1.2%, N: 246)	3 (1.2%, N: 247)
Grade 2	0 (0%, N: 246)	0 (0%, N: 247)	0 (0%, N: 246)	0 (0%, N: 247)	1 (0.4%, N: 246)	1 (0.4%, N: 247)

	Fatigue		Muscular pain	
Characteristic	Placebo, N = 250	Vaccine, N = 249	Placebo, N = 250	Vaccine, N = 249
**Day 1**				
Normal	242 (98%, N: 248)	235 (96%, N: 245)	243 (98%, N: 248)	238 (97%, N: 245)
Grade 1	4 (1.6%, N: 248)	8 (3.3%, N: 245)	3 (1.2%, N: 248)	6 (2.4%, N: 245)
Grade 2	1 (0.4%, N: 248)	2 (0.8%, N: 245)	1 (0.4%, N: 248)	1 (0.4%, N: 245)
Grade 3	1 (0.4%, N: 248)	0 (0%, N: 245)	1 (0.4%, N: 248)	0 (0%, N: 245)
**Day 2**				
Normal	241 (99%, N: 244)	233 (96%, N: 242)	242 (99%, N: 244)	237 (98%, N: 242)
Grade 1	1 (0.4%, N: 244)	7 (2.9%, N: 242)	1 (0.4%, N: 244)	4 (1.7%, N: 242)
Grade 2	2 (0.8%, N: 244)	2 (0.8%, N: 242)	1 (0.4%, N: 244)	1 (0.4%, N: 242)
**Day 3**				
Normal	240 (99%, N: 242)	241 (99%, N: 243)	239 (99%, N: 242)	242 (100%, N: 243)
Grade 1	1 (0.4%, N: 242)	2 (0.8%, N: 243)	2 (0.8%, N: 242)	1 (0.4%, N: 243)
Grade 3	1 (0.4%, N: 242)	0 (0%, N: 243)	1 (0.4%, N: 242)	0 (0%, N: 243)
**Day 4**				
Normal	241 (100%, N: 242)	241 (98%, N: 245)	239 (99%, N: 242)	244 (100%, N: 245)
Grade 1	0 (0%, N: 242)	4 (1.6%, N: 245)	2 (0.8%, N: 242)	1 (0.4%, N: 245)
Grade 3	1 (0.4%, N: 242)	0 (0%, N: 245)	1 (0.4%, N: 242)	0 (0%, N: 245)
**Day 5**				
Normal	245 (99%, N: 247)	243 (99%, N: 246)	245 (99%, N: 247)	245 (100%, N: 246)
Grade 1	1 (0.4%, N: 247)	3 (1.2%, N: 246)	2 (0.8%, N: 247)	1 (0.4%, N: 246)
Grade 2	1 (0.4%, N: 247)	0 (0%, N: 246)	0 (0%, N: 247)	0 (0%, N: 246)
**Day 6**				
Normal	243 (99%, N: 246)	245 (99%, N: 247)	245 (100%, N: 246)	247 (100%, N: 247)
Grade 1	2 (0.8%, N: 246)	1 (0.4%, N: 247)	1 (0.4%, N: 246)	0 (0%, N: 247)
Grade 2	1 (0.4%, N: 246)	1 (0.4%, N: 247)	0 (0%, N: 246)	0 (0%, N: 247)

Second injection

Figure S 5 Proportion of participants experiencing systemic adverse reactions in the six days following the 2nd injection in vaccine strengths of 2.5 × 10^6 $TCID_{50}$ (10 µg/dose) and placebo groups

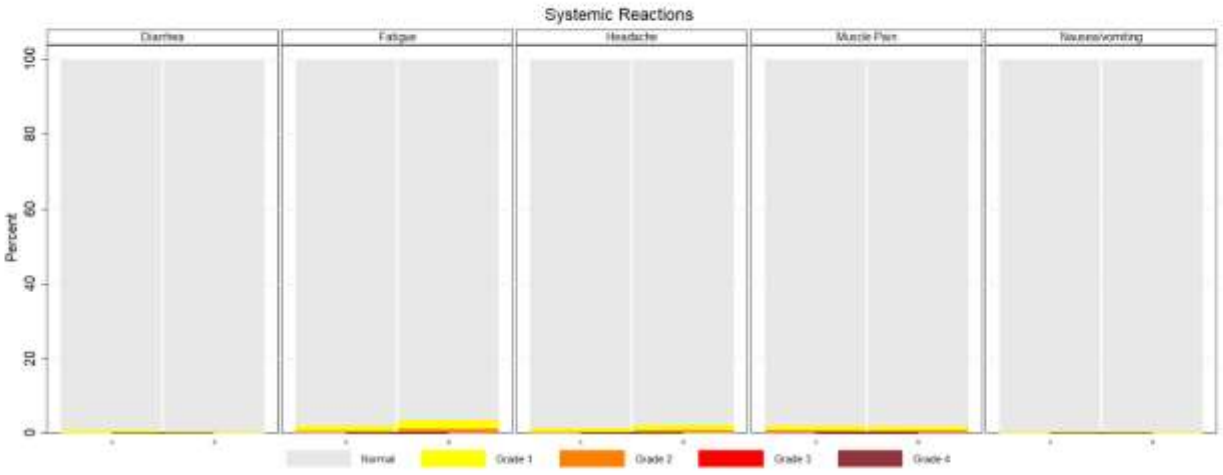

Table S 10 Grades of systemic adverse reactions in the six days following the 2nd injection in vaccine strengths of 2.5×10^6 $TCID_{50}$ (10 µg/dose) and placebo groups

	Nausea / vomiting		Diarrhea		Headache	
Characteristic	Placebo, N = 250	Vaccine, N = 249	Placebo, N = 250	Vaccine, N = 249	Placebo, N = 250	Vaccine, N = 249
**Day 15**						
Normal	210 (100%, N: 210)	206 (100%, N: 207)	208 (99%, N: 210)	207 (100%, N: 207)	207 (99%, N: 210)	206 (100%, N: 207)
Grade 1	0 (0%, N: 210)	1 (0.5%, N: 207)	2 (1.0%, N: 210)	0 (0%, N: 207)	2 (1.0%, N: 210)	1 (0.5%, N: 207)
Grade 2	0 (0%, N: 210)	0 (0%, N: 207)	0 (0%, N: 210)	0 (0%, N: 207)	1 (0.5%, N: 210)	0 (0%, N: 207)
**Day 16**						
Normal	218 (100%, N: 218)	213 (100%, N: 214)	218 (100%, N: 218)	213 (100%, N: 214)	215 (99%, N: 218)	209 (98%, N: 214)
Grade 1	0 (0%, N: 218)	1 (0.5%, N: 214)	0 (0%, N: 218)	1 (0.5%, N: 214)	3 (1.4%, N: 218)	4 (1.9%, N: 214)
Grade 2	0 (0%, N: 218)	0 (0%, N: 214)	0 (0%, N: 218)	0 (0%, N: 214)	0 (0%, N: 218)	1 (0.5%, N: 214)
**Day 17**						
Normal	217 (100%, N: 218)	213 (100%, N: 214)	216 (99%, N: 218)	213 (100%, N: 214)	216 (99%, N: 218)	214 (100%, N: 214)
Grade 1	1 (0.5%, N: 218)	1 (0.5%, N: 214)	2 (0.9%, N: 218)	1 (0.5%, N: 214)	1 (0.5%, N: 218)	0 (0%, N: 214)
Grade 2	0 (0%, N: 218)	0 (0%, N: 214)	0 (0%, N: 218)	0 (0%, N: 214)	1 (0.5%, N: 218)	0 (0%, N: 214)
**Day 18**						
Normal	217 (100%, N: 218)	211 (99%, N: 213)	217 (100%, N: 218)	213 (100%, N: 213)	216 (99%, N: 218)	207 (97%, N: 213)
Grade 1	1 (0.5%, N: 218)	2 (0.9%, N: 213)	1 (0.5%, N: 218)	0 (0%, N: 213)	1 (0.5%, N: 218)	3 (1.4%, N: 213)
Grade 2	0 (0%, N: 218)	0 (0%, N: 213)	0 (0%, N: 218)	0 (0%, N: 213)	1 (0.5%, N: 218)	3 (1.4%, N: 213)
**Day 19**						
Normal	217 (99%, N: 219)	212 (100%, N: 213)	216 (99%, N: 219)	211 (99%, N: 213)	214 (98%, N: 219)	204 (96%, N: 213)
Grade 1	2 (0.9%, N: 219)	0 (0%, N: 213)	3 (1.4%, N: 219)	2 (0.9%, N: 213)	4 (1.8%, N: 219)	7 (3.3%, N: 213)
Grade 2	0 (0%, N: 219)	1 (0.5%, N: 213)	0 (0%, N: 219)	0 (0%, N: 213)	1 (0.5%, N: 219)	2 (0.9%, N: 213)
**Day 20**						
Normal	221 (100%, N: 222)	224 (100%, N: 224)	221 (100%, N: 222)	222 (99%, N: 224)	220 (99%, N: 222)	218 (97%, N: 224)
Grade 1	1 (0.5%, N: 222)	0 (0%, N: 224)	1 (0.5%, N: 222)	2 (0.9%, N: 224)	1 (0.5%, N: 222)	3 (1.3%, N: 224)
Grade 2	0 (0%, N: 222)	0 (0%, N: 224)	0 (0%, N: 222)	0 (0%, N: 224)	1 (0.5%, N: 222)	3 (1.3%, N: 224)

	Fatigue		Muscular pain	
Characteristic	Placebo, N = 250	Vaccine, N = 249	Placebo, N = 250	Vaccine, N = 249
**Day 15**				
Normal	206 (98%, N: 210)	202 (98%, N: 207)	206 (98%, N: 210)	204 (99%, N: 207)
Grade 1	3 (1.4%, N: 210)	4 (1.9%, N: 207)	3 (1.4%, N: 210)	2 (1.0%, N: 207)
Grade 2	1 (0.5%, N: 210)	1 (0.5%, N: 207)	1 (0.5%, N: 210)	1 (0.5%, N: 207)
**Day 16**				
Normal	213 (98%, N: 218)	205 (96%, N: 214)	214 (98%, N: 218)	211 (99%, N: 214)
Grade 1	5 (2.3%, N: 218)	8 (3.7%, N: 214)	4 (1.8%, N: 218)	2 (0.9%, N: 214)
Grade 2	0 (0%, N: 218)	1 (0.5%, N: 214)	0 (0%, N: 218)	1 (0.5%, N: 214)
**Day 17**				
Normal	216 (99%, N: 218)	209 (98%, N: 214)	216 (99%, N: 218)	212 (99%, N: 214)
Grade 1	1 (0.5%, N: 218)	4 (1.9%, N: 214)	0 (0%, N: 218)	1 (0.5%, N: 214)
Grade 2	1 (0.5%, N: 218)	1 (0.5%, N: 214)	1 (0.5%, N: 218)	1 (0.5%, N: 214)
Grade 3	0 (0%, N: 218)	0 (0%, N: 214)	1 (0.5%, N: 218)	0 (0%, N: 214)
**Day 18**				
Normal	216 (99%, N: 218)	205 (96%, N: 213)	217 (100%, N: 218)	208 (98%, N: 213)
Grade 1	2 (0.9%, N: 218)	3 (1.4%, N: 213)	1 (0.5%, N: 218)	3 (1.4%, N: 213)
Grade 2	0 (0%, N: 218)	5 (2.3%, N: 213)	0 (0%, N: 218)	2 (0.9%, N: 213)
**Day 19**				
Normal	212 (97%, N: 219)	205 (96%, N: 213)	212 (97%, N: 219)	208 (98%, N: 213)
Grade 1	4 (1.8%, N: 219)	6 (2.8%, N: 213)	3 (1.4%, N: 219)	3 (1.4%, N: 213)
Grade 2	3 (1.4%, N: 219)	1 (0.5%, N: 213)	3 (1.4%, N: 219)	1 (0.5%, N: 213)
Grade 3	0 (0%, N: 219)	1 (0.5%, N: 213)	1 (0.5%, N: 219)	1 (0.5%, N: 213)
**Day 20**				
Normal	217 (98%, N: 222)	216 (96%, N: 224)	216 (97%, N: 222)	220 (98%, N: 224)
Grade 1	3 (1.4%, N: 222)	4 (1.8%, N: 224)	4 (1.8%, N: 222)	2 (0.9%, N: 224)
Grade 2	1 (0.5%, N: 222)	2 (0.9%, N: 224)	1 (0.5%, N: 222)	0 (0%, N: 224)
Grade 3	1 (0.5%, N: 222)	2 (0.9%, N: 224)	0 (0%, N: 222)	2 (0.9%, N: 224)
Grade 4	0 (0%, N: 222)	0 (0%, N: 224)	1 (0.5%, N: 222)	0 (0%, N: 224)

Laboratory findings

first injection

Table S 11 Grading of abnormal laboratory findings one week after the first injection in vaccine strengths of 2.5 × 10^6 TCID50 (10 μg/dose) and placebo groups

Characteristic	Placebo, N = 250[1]	Vaccine, N = 249[1]
**U/A RBC**		
Grade 1	10 (4.4%)	7 (3.2%)
Grade 2	4 (1.8%)	2 (0.9%)
Normal	211 (94%)	208 (96%)
**AST**		
Grade 1	3 (1.3%)	1 (0.4%)
Grade 2	2 (0.9%)	1 (0.4%)
Grade 3	1 (0.4%)	0 (0%)
Normal	227 (97%)	227 (99%)

Characteristic	Placebo, N = 250[1]	Vaccine, N = 249[1]
**ALT**		
Grade 1	9 (3.9%)	4 (1.7%)
Grade 3	1 (0.4%)	0 (0%)
Normal	223 (96%)	225 (98%)
**CPK**		
Grade 1	4 (1.7%)	3 (1.3%)
Grade 2	6 (2.6%)	1 (0.4%)
Grade 3	2 (0.9%)	1 (0.4%)
Grade 4	3 (1.3%)	0 (0%)
Normal	219 (94%)	224 (98%)
**ALK**		
Grade 1	3 (1.3%)	1 (0.4%)
Normal	230 (99%)	228 (100%)
**Bilirubin**		
Grade 1	15 (6.4%)	6 (2.6%)
Grade 2	0 (0%)	1 (0.4%)
Grade 3	0 (0%)	1 (0.4%)
Normal	218 (94%)	221 (97%)
**WBC**		
Decrease Grade 1	2 (0.9%)	0 (0%)
Decrease Grade 2	0 (0%)	1 (0.4%)
Increase Grade 1	4 (1.7%)	3 (1.3%)
Normal	227 (97%)	225 (98%)
**Neutrophils**		
Grade 1	3 (1.3%)	2 (0.9%)
Grade 3	0 (0%)	1 (0.4%)
Normal	230 (99%)	226 (99%)
**Platelets**		
Grade 1	1 (0.4%)	0 (0%)
Grade 2	1 (0.4%)	0 (0%)
Normal	231 (99%)	228 (100%)
**BUN**		
Normal	233 (100%)	229 (100%)
**Potassium**		
Hypo Grade 1	1 (0.4%)	0 (0%)
Hyper Grade 1	1 (0.4%)	0 (0%)
Normal	231 (99%)	229 (100%)
**Sodium**		
Normal	233 (100%)	229 (100%)
**Creatinine**		
Grade 1	1 (0.4%)	1 (0.4%)
Normal	232 (100%)	228 (100%)
**ESR**		
Grade 1	5 (2.1%)	3 (1.3%)
Normal	228 (98%)	226 (99%)
**CRP**		
Grade 1	3 (1.3%)	4 (1.7%)
Grade 2	1 (0.4%)	0 (0%)
Grade 3	0 (0%)	1 (0.4%)
Grade 4	1 (0.4%)	0 (0%)
Normal	228 (98%)	224 (98%)
**LDH**		
Grade 1	4 (1.7%)	0 (0%)
Grade 2	1 (0.4%)	0 (0%)
Grade 4	1 (0.4%)	0 (0%)
Normal	228 (98%)	229 (100%)

[1] n (%)

Figure S 6 Adverse laboratory events one week after the first injection

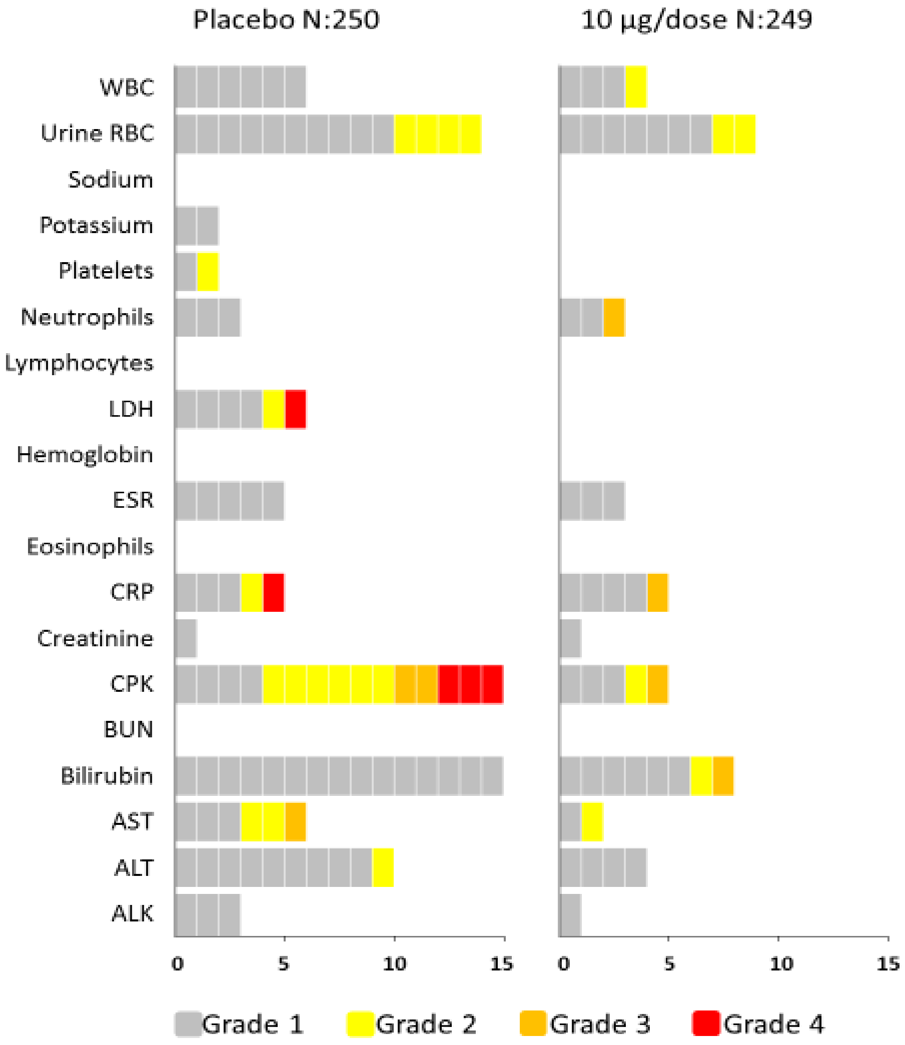

Grade I and II abnormal laboratory findings were observed in 41 and 85 vaccine and placebo recipients respectively. They included abnormal WBC [4 (0.016%) and 6 [(0.024%)], Neutrophils [2 (0.008%) and 3 (0.012%)], ESR[3 (0.012%) and 5 (0.02%)], CRP [4 (0.016%) and 4 (0.016%)], Creatinine [1(0.004%) and 1(0.004%)], CPK [4(0.016%) and 10 (0.04%)], Bilirubin [7 (0.028%) and 15 (0.06%)], AST [2 (0.008%) and 5 (0.02%)], ALT [4 (.016%) and 10 (0.04%)], ALK [1 (0.004%) and 3 (0.012%)] and Urine RBC [9 (0.04%) and 14 (0.056%)]. Of the 7 grade III abnormalities seen, 4 (Abnormal Neutrophils count [1 (0.004%)], CRP [1 (0.004%)], CPK [1 (0.004%)] and Bilirubin [1 (0.004%)]) occurred in the vaccine group and 3 (Abnormal CPK count [2 (0.008%)] and AST [1 (0.004%)]) in the placebo group.

Table S 12 Changes in laboratories indices of a 43 years old man receiving the placebo

Day	CPK	LDH
0	102 (Normal)	247 (Normal)
7	13320 (Grade 4)	1219 (Grade 4)
9	1647 (Grade 3)	285 (Normal)
13	461 (Grade 2)	266 (Normal)
20	240 (Normal)	295 (Normal)
24	149 (Normal)	264 (Normal)

Table S 13 Changes in laboratories indices of a 29 years old man receiving the placebo

Day	CPK
0	137.1 (Normal)
7	4361 (Grade 4)
10	1722 (Grade 3)
13	1110 (Grade 3)
15	594 (Grade 3)
18	336 (Normal)
23	229 (Normal)

Table S 14 Changes in laboratories indices of a 33 years old man receiving the placebo

Day	CPK
0	86.7 (Normal)
7	3265 (Grade 4)
9	4200 (Grade 4)
11	4380 (Grade 4)

15	461 (Normal)
17	260 (Normal)
25	94 (Normal)

Table S 15 Changes in laboratories indices of a 22 years old man receiving the placebo

Day	CRP
0	0.3 (Normal)
7	52.6 (Grade 4)
14	1.7 (Normal)

second injection

Table S 16 Grading of abnormal laboratory findings one week after the second injection in vaccine strengths of 2.5 × 10^{6} TCID50 (10 µg/dose) and placebo groups

Characteristic	Placebo, N = 12[1]	Vaccine, N = 13[1]
**U/A RBC**		
Normal	9 (100%)	8 (100%)
**AST**		
Normal	10 (100%)	9 (100%)
**ALT**		
Normal	10 (100%)	9 (100%)
**CPK**		
Normal	10 (100%)	9 (100%)
**ALK**		
Normal	10 (100%)	9 (100%)
**Bilirubin**		
Normal	10 (100%)	9 (100%)
**WBC**		
Normal	9 (100%)	9 (100%)
**Neutrophils**		
Normal	9 (100%)	9 (100%)
**Platelets**		
Normal	9 (100%)	9 (100%)
**BUN**		
Normal	9 (100%)	8 (90%)
Grade 1	0 (0%)	1 (10%)
**Potassium**		
Normal	9 (100%)	9 (100%)
**Sodium**		

Characteristic	Placebo, N = 12[1]	Vaccine, N = 13[1]
Normal	9 (100%)	8 (100%)
**Creatinine**		
Normal	9 (100%)	9 (100%)
**ESR**		
Normal	9 (100%)	9 (100%)
**CRP**		
Normal	9 (100%)	9 (100%)
**LDH**		
Normal	10 (100%)	9 (100%)

[1]n (%)

Immunological indices

Peripheral blood flowcytometry for lymphocyte subtypes composition

Figure S 7 Proportion of lymphocytic population expressing CD3 marker in peripheral blood measured by flowcytometry at day 0 and one week after first injection in study groups (A:Placebo; B: Vaccine)

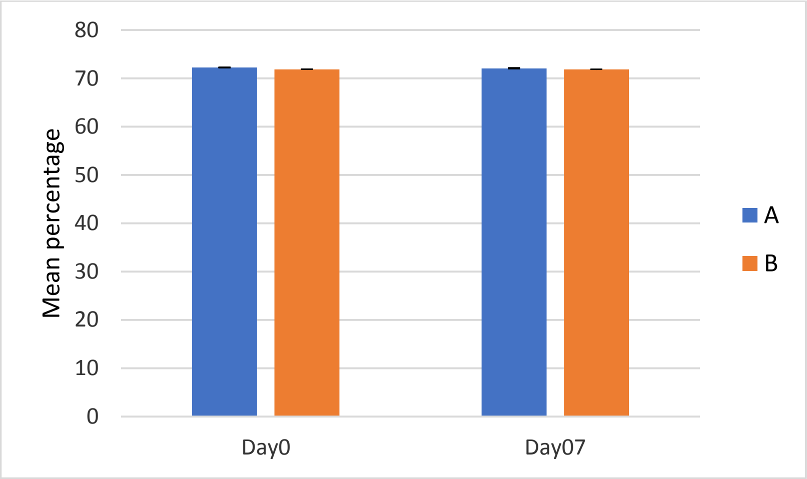

Figure S 8 Proportion of lymphocytic population expressing CD4 marker within CD3 positive subset in peripheral blood measured by flowcytometry at day 0 and one week after first injection in study groups (A:Placebo; B: Vaccine)

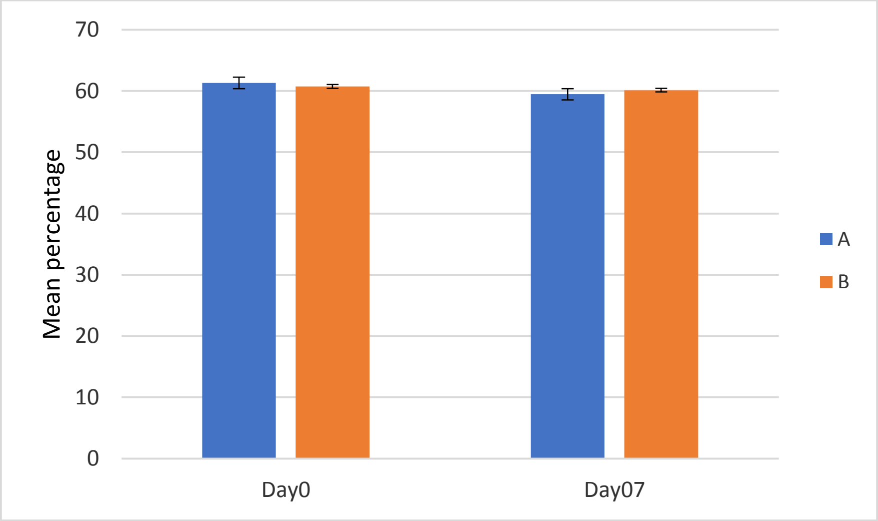

Figure S 9 Proportion of lymphocytic population expressing CD8 marker within CD3 positive subset in peripheral blood measured by flowcytometry at day 0 and one week after first injection in study groups (A:Placebo; B: Vaccine)

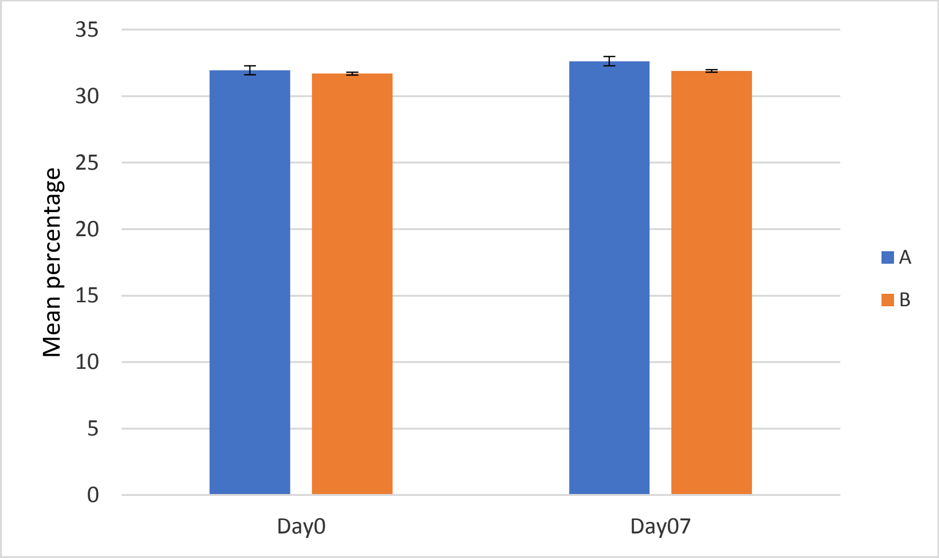

Figure S 10 CD4/CD8 ratio within CD3 positive lymphocytic subset in peripheral blood measured by flowcytometry at day 0 and one week after first injection in study groups (A:Placebo; B: Vaccine)

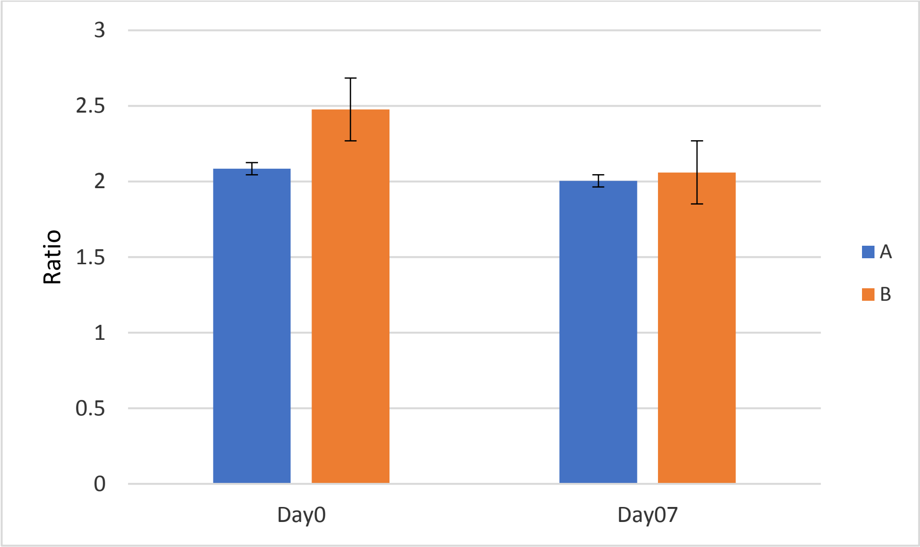

Figure S 11 CD4/CD8 ratio within lymphocytic cell population in peripheral blood measured by flowcytometry at day 0 and one week after first injection in study groups (A:Placebo; B: Vaccine)

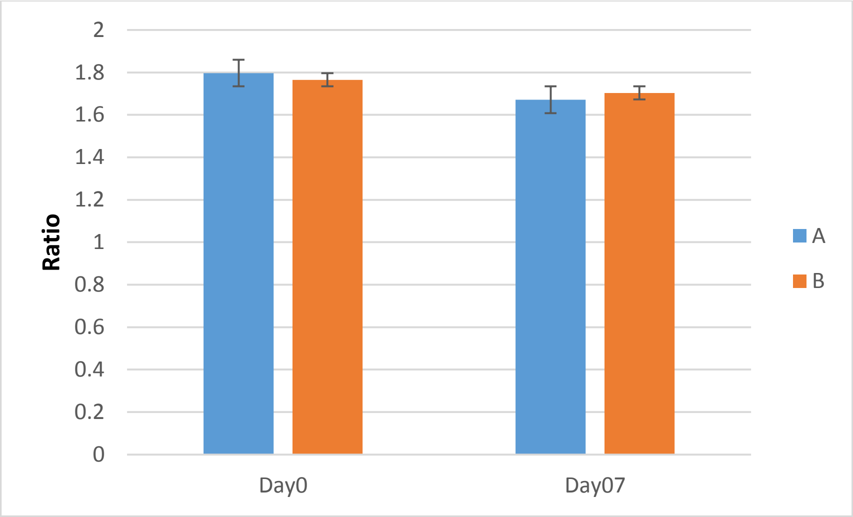

Figure S 12 Proportion of lymphocytic population expressing CD56 marker within CD3 negative subset in peripheral blood measured by flowcytometry at day 0 and one week after first injection in study groups *(A:Placebo; B: Vaccine)*

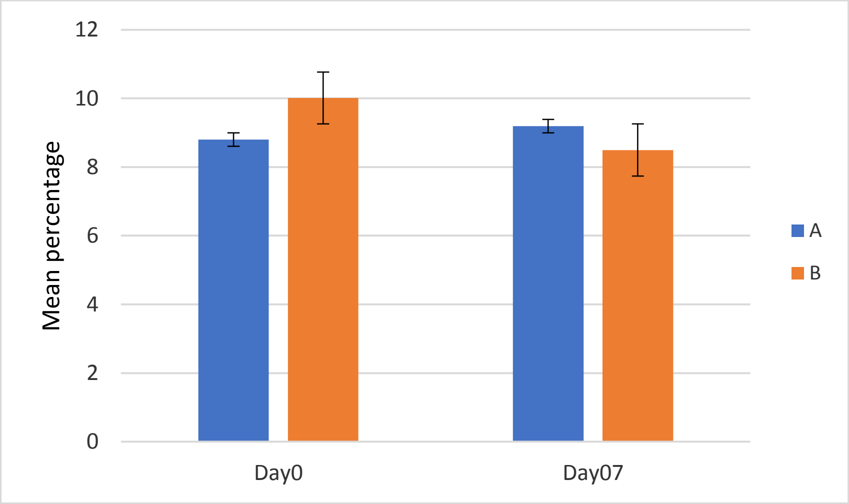

Figure S 13 Proportion of CD19 or CD20 in peripheral blood measured by flowcytometry at day 0 and one week after first injection in study groups (A:Placebo; B: Vaccine)

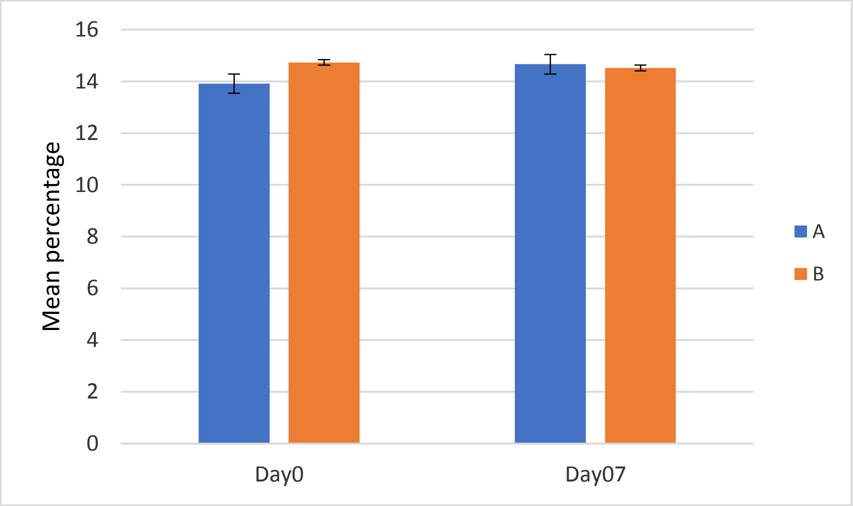

Figure S 14 Serum concentrations pg/ml of IL-6 in study participants at day 0 and 14 days after 2nd injection in two administration schedules and vaccine strength of 2.5 × 10^6 $TCID_{50}$ (10 µg/dose) and placebo

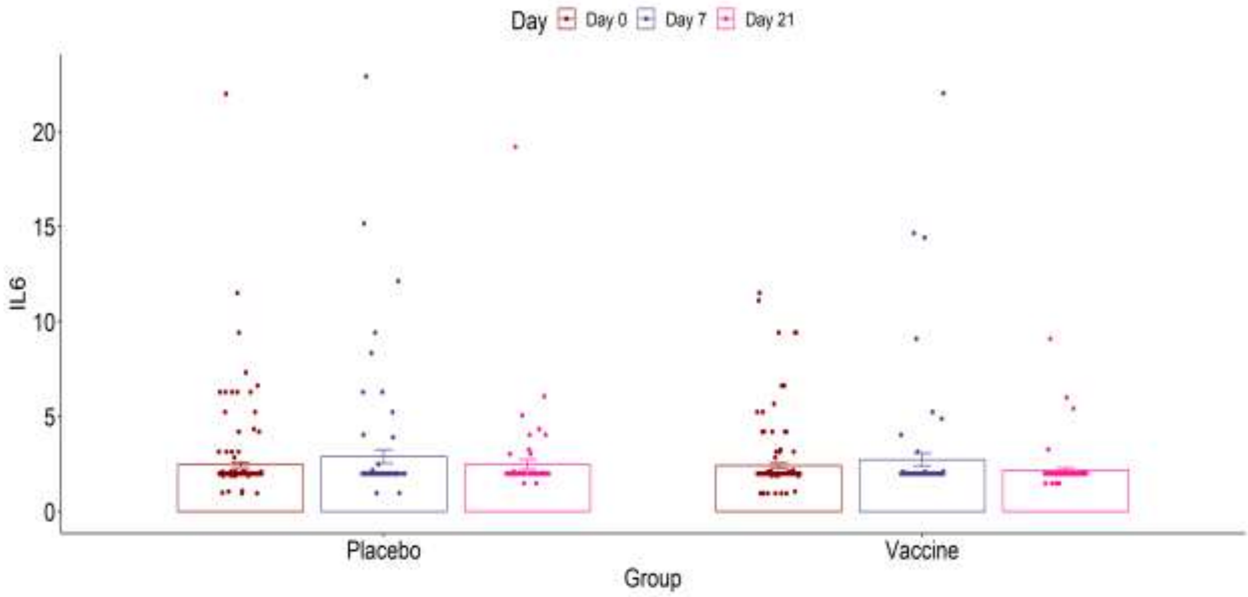

immunogenicity outcomes

Table S 17 Geometric mean titer, geometric mean ratio (compared to placebo), and geometric mean fold increase (compared to day zero) for serum neutralizing antibody titers and their 95% confidence intervals at predefined time intervals in study groups

day	0	14	28	42	90	180
**GMT**						
Placebo	1.52 (1.37-1.70, N:250)	1.48 (0.93-2.35, N:16)	1.72 (1.48-1.99, N:151)	2.26 (1.85-2.75, N:142)	2.45 (0-215154, N:2)	-
Vaccine	1.31 (1.21-1.43,, N:249)	2.16 (1.31-3.58, N:32)	5.90 (4.48-7.78, N:158)	11.88 (9.34-15.10, N:143)	11.73 (4.01-34.33, N:10)	24.60 (14.79-40.92, N:65)
**GMR**						
Placebo	1	1	1	1	1	-
Vaccine	0.85 (0.44-1.67, N:249)	1.46 (0.56-6.84, N:16)	2.69 (2.32-3.12, N:151)	5.51 (3.94-8.35, N:142)	5.02 (2.04-314.44, N:2)	-
**GMFI**						
Placebo	1	1.09 (0.15-7.81, N:16)	1.10 (0.99-1.21, N:151)	1.49 (0.95-2.64, N:142)	1.37 (0.09-22.51, N:2)	-
Vaccine	1	2.83 (1.25-5.73, N:32)	4.62 (3.62-5.90, N:158)	9.13 (7.68-12.81, N:143)	9.05 (5.56-15.92, N:10)	18.57 (15.13-28.97, N:65)
**GMFR**						
Placebo	1	1		1	1	-
Vaccine	1	1.29 (0.76-3.12, N:16)	7.54 (9.87-19.91, N:158)	3.06 (2.26-5.05, N: 142)	3.30 (1.66-7.11, N:2)	-

Neutralizing antibody titers in the blood are closely correlated with protection provided by an effective vaccination. As a gold standard for assessing protective antibodies, the virus neutralization test (VNT) measures the ability of these antibodies to neutralize infectivity and protect cells from infection. This study used the Vero E6 cell line for virus propagation. Therefore, Vero E6 cells were cultured in DMEM medium containing 10% FBS, and then infection and replication of the SARS-CoV-2 virus were performed. The VNT test based on Spearman and Kairber's method and based on the 50% Tissue Culture Infectious Dose (TCID50) was performed as follows. At first, a volume of 200 µl of serial-diluted serum (1, 1.2, 1.4, 1.8, 1.16, 1.32, 1.64, 1.128, and 1.256) was prepared in the DMEM medium without FBS. For each serum, four wells were considered in a sterile flat-bottom 96-well plate. Then 200 µl of 400 TCID50 per ml of SARS-CoV-2 was added to each serum and was incubated at 37 °C for 2 hours. The inoculum was removed, infected cells were washed once with 5% FBS in DMEM, and 100 µl of serum-virus suspension was transferred to each of them. The final dilutions included 1.2, 1.4, 1.8, 1.16, 1.32, 1.64, 1.128, 1.256, and 1.512 of the serum sample. In this study, positive and negative controls, including cell-free culture medium and virus-containing culture medium, were considered. Then the microplates were incubated at 36°C and 5% CO_2 for three days. The plates were checked daily by microscopic observation. Finally, the ability to neutralize sera antibodies was evaluated against the virus.

Neutralization antibody activity

Table S 18 The proportion of participants with a four-fold increase in neutralizing antibody titer at predefined time intervals in vaccine strengths of 2.5 × 10^6 TCID50 (10 µg/dose) and placebo groups at day 42

	Numbers without four-fold increase	Numbers with four-fold increase	Total
**Placebo**	119<br>83.80%	23<br>16.20%	142<br>100%
**Vaccine**	138<br>26.58%	105<br>73.42%	143<br>100%
**Total**	157<br>55.09%	128<br>44.91%	285<br>100%

Table S 19 The proportion of participants with a four-fold increase in neutralizing antibody titer at predefined time intervals in vaccine strengths of 2.5 × 10^6 TCID50 (10 µg/dose) and placebo groups at day 90

	Numbers without four-fold increase	Numbers with four-fold increase	Total
**Placebo**	1<br>50%	1<br>50%	2<br>100%
**Vaccine**	3<br>30%	7<br>70%	10<br>100%
**Total**	4<br>33.33%	8<br>66.67%	12<br>100%

Table S 20 The proportion of participants with a four-fold increase in neutralizing antibody titer at predefined time intervals in vaccine strengths of 2.5 × 10^6 TCID50 (10 µg/dose) and placebo groups at day 180

	Numbers without four-fold increase	Numbers with four-fold increase	Total
**Placebo**	–	–	–
**Vaccine**	21<br>33.87%	41<br>66.13%	62<br>100%

**Total**	21<br>33.87%	41<br>66.13%	62<br>100%

Figure S 15 Neutralizing antibodies at different time points among two groups

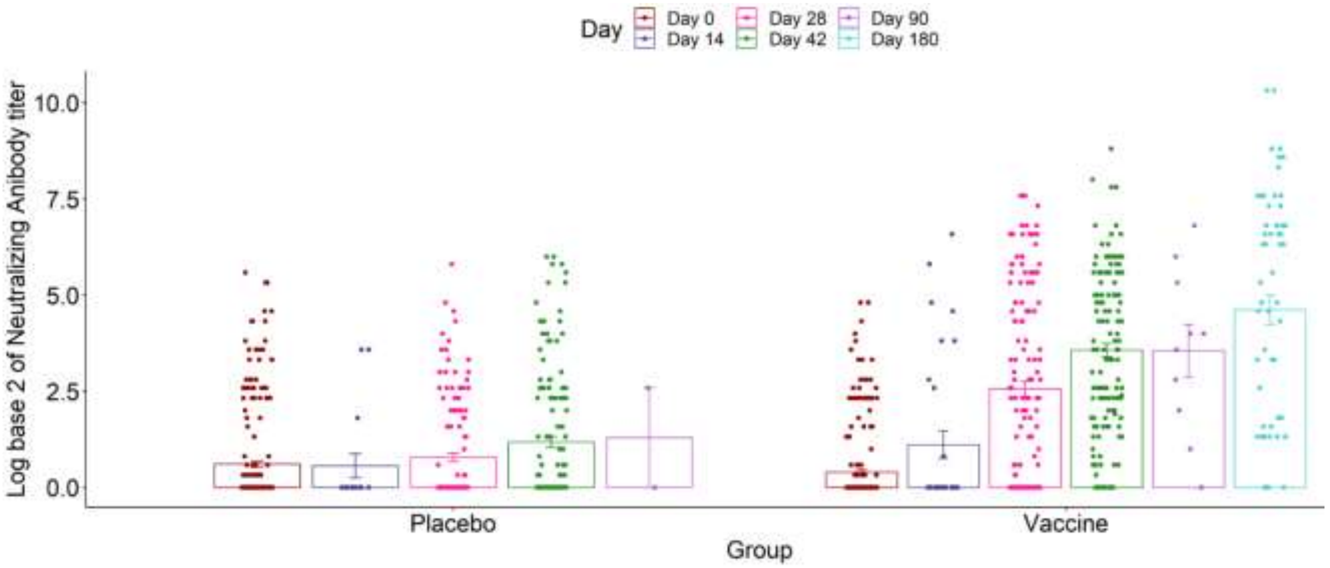

Serum ELISA IgG levels for SARS-CoV-2

Serum IgG levels for S1RBD antigen

Table S 21 Geometric mean titer, geometric mean ratio (compared to placebo), and geometric mean fold increase (compared to day zero) for serum specific IgG antibody levels against S1RBD antigen and their 95% confidence intervals at predefined time intervals in study groups

Day	0	14	28	42	90	180
**GM**						
Placebo	0.42 (0.38-0.47, N:250)	0.44 (0.4-0.5, N:229)	0.47 (0.41-0.53, N:193)	0.68 (0.55-0.84, N:143)	0.68 (0.46-1, N:40)	-
Vaccine	0.38 (0.34-0.42, N:249)	0.52 (0.45-0.6, N:229)	1.08 (0.92-1.27, N:193)	2.28 (1.85-2.8, N:142)	1.63 (1.27-2.1, N:77)	1.49 (1.11-2, N:74)
**GMR**						
Placebo	1	1	1	1	1	-
Vaccine	0.9 (0.78-1.05, N:249)	1.17 (0.97-1.4, N:229)	2.31 (2.03-2.62, N:193)	3.34 (2.5-4.47, N:142)	2.39 (1.54-3.73, N:40)	-
**GMFI**						
Placebo	1	1.09 (1.02-1.16, N:229)	1.08 (0.99-1.18, N:193)	1.6 (1.37-1.87, N:143)	1.36 (1.01-1.84, N:40)	-
Vaccine	1	1.37 (1.25-1.5, N:229)	2.89 (2.55-3.27, N:193)	5.75 (4.71-7.02, N:142)	3.61 (2.93-4.43, N:77)	3.65 (2.79-4.78, N:74)
**GMFR**						
Placebo	1	1	1	1	1	-
Vaccine	1	3.61 (3-4.36, N:229)	3.6 3.06-4.23, N:193)	2.59 (1.95-3.43, N:142)	1.27 (1.08-1.48, N:40)	-

Antibody detection assays for SARS-CoV-2 are essential for gathering serological information and monitoring antibody responses. Spike protein's receptor-binding domain (S1RBD) is a particular target for detecting anti-SARS-CoV-2 antibodies among the types of SARS-CoV-2 antigens. In order to understand antibody-mediated protection and vaccine response, it is essential to detect IgG antibodies against S1RBD. Therefore, in this study, we used a rapid and stable RBD-based IgG ELISA test Kit (Anti-SARS-CoV-2 S1RBD (IgG), DiaZist, DCVS 002A, commercial kit). For normalization, the optical density (OD) of the samples at 450 nm was subtracted from the OD of the negative test control. Finally, the results were calculated as an index using the kit's formula. Based on the formula, values higher than 1.1 were positive, and values lower than 0.9 were reported as negative.

Cut-off index (COI) = (OD of sample-OD of negative control)/OD of cut-off value

Figure S 16 Changes in serum specific IgG antibody levels against S1RBD antigen over the study period for each individual participant and the group mean in vaccine strengths of 2.5 × 10^6 $TCID_{50}$ (10 µg/dose) and placebo groups

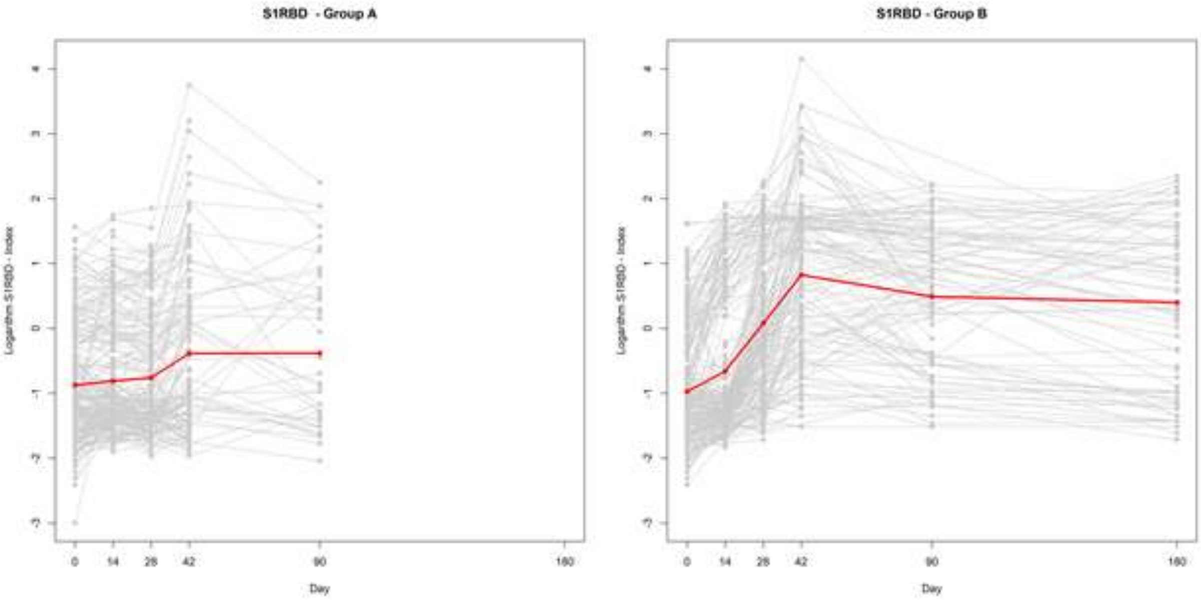

Figure S 17 Changes in group means of serum specific IgG antibody levels against S1RBD antigen over the study period in vaccine strengths of 2.5 × 10^6 $TCID_{50}$ (10 µg/dose) and placebo groups

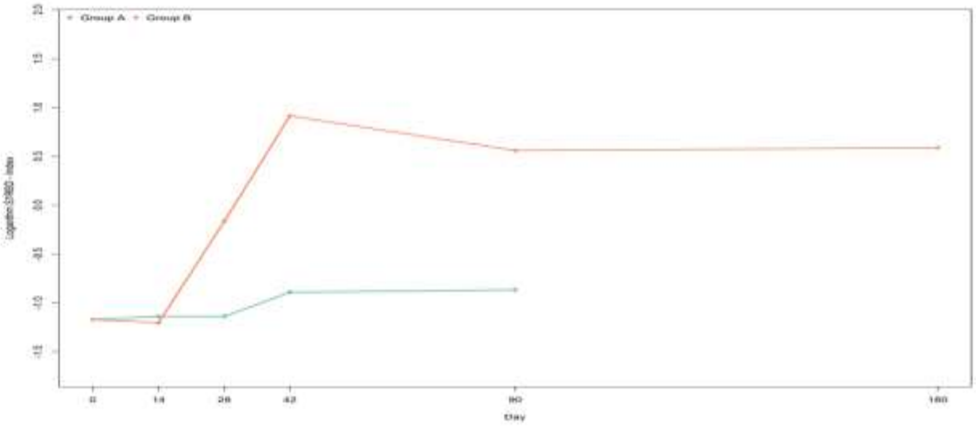

Figure S 18 Serum specific IgG antibody levels against S1RBD antigen over the study period for each individual participant and the group mean and its 95% confidence interval in vaccine strengths of 2.5 × 10^6 $TCID_{50}$ (10 µg/dose) and placebo groups

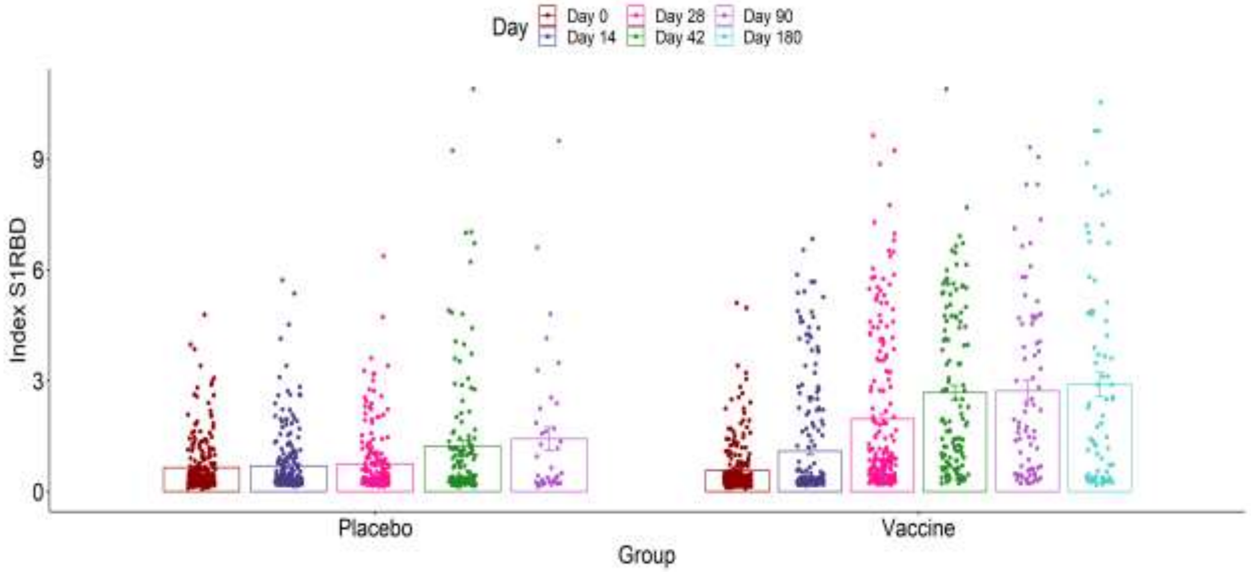

Serum IgG levels for Nucleocapsid antigen

Table S 22 Geometric mean, geometric mean ratio (compared to placebo), geometric mean fold increase (compared to day zero), and geometric mean fold ratio for serum specific IgG antibody levels against N antigen and their 95% confidence intervals at predefined time intervals in vaccine strengths of 2.5 × 10^6 $TCID_{50}$ (10 µg/dose) and placebo groups

day	0	14	28	42	90	180
**GM**						
Placebo	0.59 (0.55-0.64, N:249)	0.53 (0.48-0.58, N:229)	0.46 (0.42-0.51, N:193)	0.66 (0.6-0.72, N:143)	1.09 (0.79-1.5, N:40)	-
Vaccine	0.57 (0.53-0.61, N:249)	0.75 (0.66-0.86, N:229)	1.59 (1.36-1.86, N:193)	2 (1.65-2.41, N:142)	2.35 (1.9-2.92, N:77)	1.25 (0.97-1.6, N:74)
**GMR**						
Placebo	1	1	1	1	1	-
Vaccine	0.96 (0.86-1.07, N:249)	1.43 (1.22-1.67, N:229)	3.43 (3.10-3.79, N:193)	3.04 (2.46-3.75, N:142)	2.16 (1.48-3.13, N:142)	-
**GMFI**						
Placebo	1	0.9 (0.82-0.99, N:228)	0.75 (0.68-0.84, N:192)	1.13 (1.02-1.25, N:142)	1.81 (1.28-2.57, N:40)	-
Vaccine	1	1.32 (1.16-1.5, N:229)	2.84 (2.43-3.32, N:193)	3.7 (3.06-4.47, N:142)	4.03 (3.23-5.02, N:77)	2.09 (1.6-2.73, N:74)
**GMFR**						
Placebo	1	1	1	1	1	-
Vaccine	1	3.2 (2.65-3.87, N:229)	3.18 (2.64-3.85, N:193)	2.17 (1.63-2.88, N:142)	1.48 (1.26-1.74, N:77)	-

Figure S 19 Changes in serum specific IgG antibody levels against N antigen over the study period for each individual participant and the group mean in vaccine strengths of 2.5 × 10^6 $TCID_{50}$ (10 µg/dose) and placebo groups

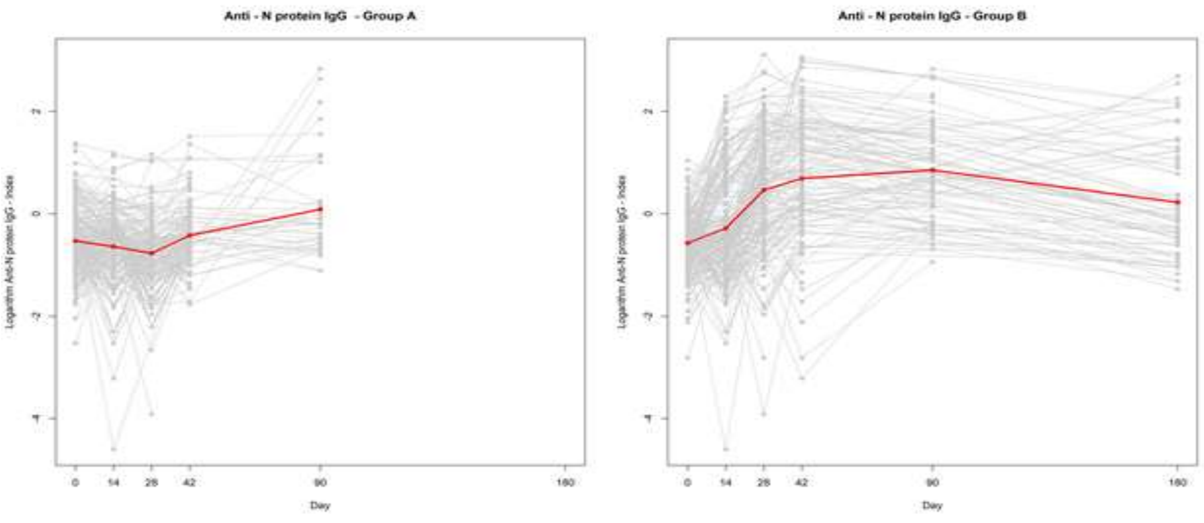

Figure S 20 Changes in group means of serum specific IgG antibody levels against N antigen over the study period in vaccine strengths of 2.5×10^6 $TCID_{50}$ (10 µg/dose) and placebo groups

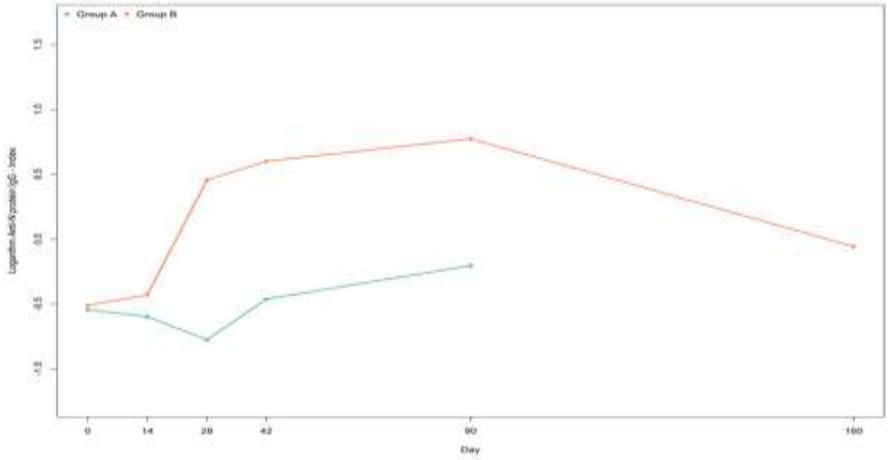

Figure S 21 Serum specific IgG antibody levels against N antigen over the study period for each individual participant and the group mean and its 95% confidence interval in vaccine strengths of 2.5×10^6 $TCID_{50}$ (10 µg/dose) and placebo groups

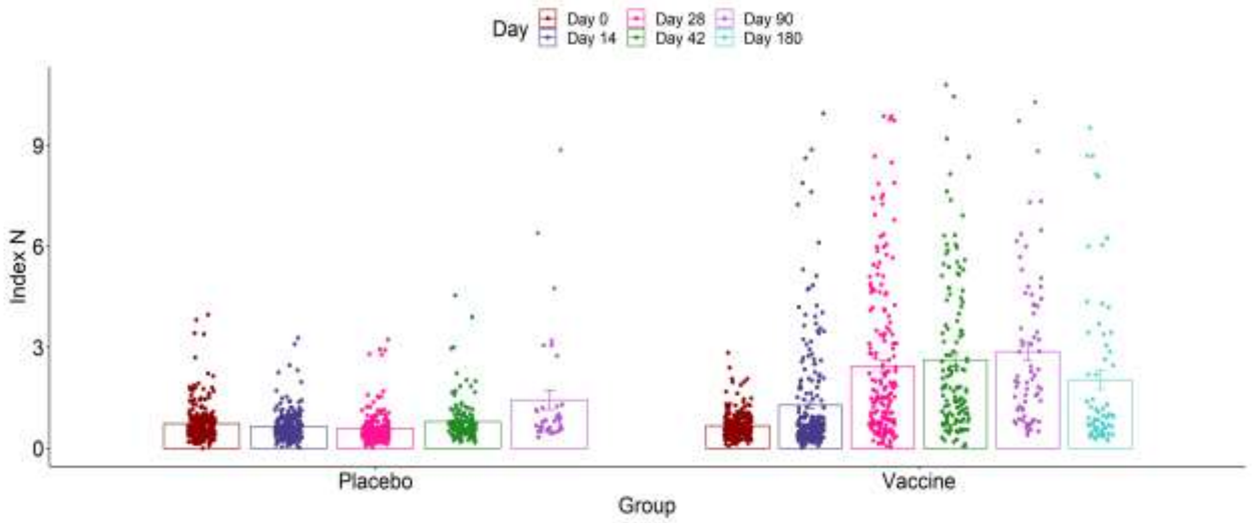

Withdrawal cases

Table S 23 Dates and reasons for withdrawals in the study participants

Study Group	First vaccine date	Second vaccine date	Withdrawal date	Reason for withdrawal
Placebo	06/09/2021	06/23/2021	08/11/2021	Received another vaccine
Placebo	06/09/2021	06/23/2021	07/24/2021	Received another vaccine
Vaccine	06/09/2021	06/23/2021	07/07/2021	Contracted Covid 19
Placebo	06/09/2021	06/23/2021	09/12/2021	Received another vaccine
Placebo	06/09/2021	06/23/2021	08/03/2021	Received another vaccine
Placebo	06/09/2021	06/23/2021	08/05/2021	Received another vaccine
Vaccine	06/09/2021	06/23/2021	08/05/2021	Received another vaccine
Placebo	06/09/2021	06/23/2021	08/06/2021	Received another vaccine
Vaccine	06/09/2021	0000/00/00	06/16/2021	Contracted Covid 19
Placebo	06/09/2021	06/23/2021	09/07/2021	Received another vaccine
Placebo	06/09/2021	06/24/2021	09/09/2021	Received another vaccine
Vaccine	06/09/2021	06/24/2021	08/11/2021	Contracted Covid 19
Vaccine	06/09/2021	06/23/2021	09/11/2021	Received another vaccine
Vaccine	06/10/2021	06/24/2021	08/01/2021	Contracted Covid 19
Placebo	06/10/2021	06/24/2021	08/04/2021	Received another vaccine
Vaccine	06/10/2021	06/24/2021	07/01/2021	Contracted Covid 19
Placebo	06/10/2021	06/24/2021	08/14/2021	Received another vaccine
Vaccine	06/10/2021	06/24/2021	08/20/2021	Received another vaccine
Vaccine	06/10/2021	06/24/2021	07/01/2021	Contracted Covid 19
Placebo	06/10/2021	06/26/2021	08/15/2021	Contracted Covid 19
Vaccine	06/10/2021	06/24/2021	07/28/2021	Received another vaccine
Placebo	06/10/2021	06/26/2021	08/13/2021	Received another vaccine
Vaccine	06/10/2021	06/24/2021	07/08/2021	Contracted Covid 19
Placebo	06/10/2021	06/24/2021	08/26/2021	Received another vaccine
Vaccine	06/10/2021	06/24/2021	08/12/2021	Received another vaccine
Placebo	06/10/2021	06/24/2021	09/12/2021	Received another vaccine
Vaccine	06/10/2021	06/26/2021	07/01/2021	Contracted Covid 19
Placebo	06/10/2021	06/28/2021	08/12/2021	Received another vaccine
Placebo	06/10/2021	0000/00/00	06/10/2021	Contracted Covid 19
Vaccine	06/10/2021	06/24/2021	08/26/2021	Contracted Covid 19
Placebo	06/10/2021	06/24/2021	07/01/2021	Contracted Covid 19
Placebo	06/10/2021	06/24/2021	07/08/2021	Contracted Covid 19
Placebo	06/10/2021	06/24/2021	07/30/2021	Received another vaccine

Placebo	06/10/2021	06/24/2021	08/22/2021	Received another vaccine
Vaccine	06/10/2021	06/28/2021	07/15/2021	Contracted Covid 19
Placebo	06/12/2021	06/26/2021	09/11/2021	Received another vaccine
Vaccine	06/12/2021	06/26/2021	08/27/2021	Contracted Covid 19
Placebo	06/12/2021	0000/00/00	06/12/2021	Contracted Covid 19
Vaccine	06/12/2021	06/26/2021	09/14/2021	Received another vaccine
Placebo	06/12/2021	06/26/2021	08/26/2021	Contracted Covid 19
Placebo	06/12/2021	06/26/2021	09/08/2021	Received another vaccine
Placebo	06/12/2021	06/26/2021	07/10/2021	Contracted Covid 19
Placebo	06/12/2021	06/26/2021	07/06/2021	Received another vaccine
Vaccine	06/12/2021	06/26/2021	08/06/2021	Received another vaccine
Placebo	06/12/2021	06/26/2021	07/03/2021	Contracted Covid 19
Vaccine	06/12/2021	0000/00/00	06/12/2021	Contracted Covid 19
Vaccine	06/12/2021	0000/00/00	06/12/2021	Contracted Covid 19
Placebo	06/12/2021	06/26/2021	07/03/2021	Contracted Covid 19
Vaccine	06/12/2021	06/26/2021	08/16/2021	Contracted Covid 19
Vaccine	06/12/2021	0000/00/00	06/12/2021	Contracted Covid 19
Placebo	06/12/2021	06/26/2021	07/17/2021	Loss to follow-up
Placebo	06/12/2021	0000/00/00	06/19/2021	Abnormal lab test
Placebo	06/12/2021	06/26/2021	08/10/2021	Contracted Covid 19
Placebo	06/12/2021	05/26/2021	08/23/2021	Received another vaccine
Placebo	06/12/2021	06/26/2021	08/15/2021	Received another vaccine
Vaccine	06/12/2021	06/26/2021	06/26/2021	Contracted Covid 19
Vaccine	06/12/2021	06/26/2021	07/17/2021	Loss to follow-up
Vaccine	06/12/2021	06/28/2021	07/19/2021	Loss to follow-up
Placebo	06/12/2021	06/26/2021	07/28/2021	Contracted Covid 19
Vaccine	06/12/2021	06/26/2021	08/04/2021	Received another vaccine
Placebo	06/12/2021	06/26/2021	08/11/2021	Received another vaccine
Placebo	06/12/2021	06/26/2021	07/03/2021	Contracted Covid 19
Placebo	06/12/2021	06/26/2021	08/21/2021	Contracted Covid 19
Placebo	06/12/2021	06/26/2021	07/14/2021	Contracted Covid 19
Vaccine	06/12/2021	06/26/2021	08/23/2021	Received another vaccine
Placebo	06/12/2021	07/15/2021	08/05/2021	Loss to follow-up
Placebo	06/12/2021	06/26/2021	07/25/2021	Contracted Covid 19
Vaccine	06/12/2021	06/26/2021	08/10/2021	Contracted Covid 19
Placebo	06/12/2021	06/26/2021	08/19/2021	Received another vaccine
Vaccine	06/12/2021	06/26/2021	07/03/2021	Contracted Covid 19
Vaccine	06/12/2021	06/26/2021	07/04/2021	Contracted Covid 19
Vaccine	06/12/2021	06/26/2021	07/17/2021	Loss to follow-up
Placebo	06/12/2021	0000/00/00	06/20/2021	Abnormal lab test

Vaccine	06/12/2021	06/26/2021	07/17/2021	Loss to follow-up
Placebo	06/12/2021	06/26/2021	07/03/2021	Contracted Covid 19
Vaccine	06/12/2021	06/26/2021	09/07/2021	Contracted Covid 19
Vaccine	06/12/2021	06/26/2021	07/17/2021	Loss to follow-up
Vaccine	06/12/2021	06/26/2021	07/10/2021	Contracted Covid 19
Vaccine	06/12/2021	06/26/2021	09/09/2021	Received another vaccine
Placebo	06/12/2021	0000/00/00	06/20/2021	Abnormal lab test
Placebo	06/12/2021	06/26/2021	09/13/2021	Received another vaccine
Placebo	06/12/2021	06/26/2021	09/13/2021	Received another vaccine
Placebo	06/12/2021	06/26/2021	07/10/2021	Contracted Covid 19
Placebo	06/12/2021	06/26/2021	08/14/2021	Contracted Covid 19
Placebo	06/12/2021	06/26/2021	08/17/2021	Received another vaccine
Vaccine	06/12/2021	06/26/2021	06/26/2021	Contracted Covid 19
Vaccine	06/12/2021	06/26/2021	07/17/2021	Loss to follow-up
Vaccine	06/12/2021	06/26/2021	06/26/2021	Contracted Covid 19
Vaccine	06/12/2021	06/26/2021	07/17/2021	Loss to follow-up
Vaccine	06/14/2021	06/28/2021	08/24/2021	Received another vaccine
Placebo	06/14/2021	06/28/2021	08/01/2021	Contracted Covid 19
Placebo	06/14/2021	06/28/2021	06/28/2021	Contracted Covid 19
Placebo	06/14/2021	06/28/2021	07/19/2021	Loss to follow-up
Placebo	06/14/2021	0000/00/00	06/21/2021	Abnormal lab test
Vaccine	06/14/2021	0000/00/00	06/21/2021	Contracted Covid 19
Vaccine	06/14/2021	0000/00/00	06/21/2021	Abnormal lab test
Placebo	06/14/2021	06/28/2021	07/19/2021	Loss to follow-up
Placebo	06/14/2021	06/28/2021	09/15/2021	Received another vaccine
Vaccine	06/14/2021	0000/00/00	06/14/2021	Contracted Covid 19
Vaccine	06/14/2021	06/28/2021	08/12/2021	Received another vaccine
Placebo	06/16/2021	06/30/2021	07/21/2021	Loss to follow-up
Vaccine	06/16/2021	06/30/2021	07/24/2021	Contracted Covid 19
Vaccine	06/16/2021	07/03/2021	07/24/2021	Loss to follow-up
Placebo	06/16/2021	06/30/2021	07/15/2021	Contracted Covid 19
Vaccine	06/16/2021	06/30/2021	07/14/2021	Contracted Covid 19
Placebo	06/19/2021	07/03/2021	07/03/2021	Contracted Covid 19
Placebo	06/19/2021	07/03/2021	07/24/2021	Loss to follow-up
Vaccine	06/19/2021	07/03/2021	07/07/2021	Contracted Covid 19
Vaccine	06/19/2021	07/11/2021	08/01/2021	Loss to follow-up
Placebo	06/19/2021	07/03/2021	08/18/2021	Received another vaccine
Placebo	06/19/2021	07/03/2021	07/28/2021	Contracted Covid 19
Vaccine	06/19/2021	07/03/2021	08/23/2021	Contracted Covid 19
Vaccine	06/19/2021	07/03/2021	07/17/2021	Contracted Covid 19

IUM CTC
مرکز کارآزمایی بالینی دانشگاه علوم پزشکی ایران

میلاد دارونور

Vaccine	06/19/2021	07/03/2021	07/13/2021	Contracted Covid 19
Vaccine	06/19/2021	07/03/2021	07/07/2021	Contracted Covid 19
Vaccine	06/19/2021	07/03/2021	07/24/2021	Loss to follow-up
Placebo	06/19/2021	07/03/2021	08/01/2021	Loss to follow-up
Placebo	06/19/2021	07/03/2021	07/10/2021	Contracted Covid 19
Vaccine	06/19/2021	07/03/2021	08/17/2021	Received another vaccine
Placebo	06/19/2021	07/03/2021	07/09/2021	Contracted Covid 19
Placebo	06/19/2021	07/03/2021	08/01/2021	Loss to follow-up
Vaccine	06/19/2021	07/03/2021	07/10/2021	Contracted Covid 19
Vaccine	06/21/2021	07/06/2021	07/11/2021	Contracted Covid 19
Vaccine	06/21/2021	07/06/2021	07/27/2021	Loss to follow-up
Placebo	06/21/2021	07/06/2021	08/01/2021	Contracted Covid 19
Vaccine	06/21/2021	07/12/2021	08/01/2021	Loss to follow-up
Vaccine	06/21/2021	07/08/2021	07/29/2021	Loss to follow-up
Placebo	06/21/2021	07/06/2021	08/15/2021	Received another vaccine
Placebo	06/24/2021	07/08/2021	07/24/2021	Contracted Covid 19
Placebo	06/24/2021	07/08/2021	08/06/2021	Received another vaccine
Vaccine	06/24/2021	07/08/2021	09/12/2021	Received another vaccine
Vaccine	06/24/2021	07/08/2021	07/29/2021	Loss to follow-up
Vaccine	06/24/2021	07/08/2021	08/16/2021	Contracted Covid 19
Placebo	06/24/2021	07/10/2021	07/15/2021	Contracted Covid 19
Placebo	06/24/2021	0000/00/00	07/01/2021	Contracted Covid 19
Placebo	06/24/2021	07/11/2021	07/11/2021	Contracted Covid 19
Placebo	06/24/2021	07/11/2021	08/01/2021	Loss to follow-up
Placebo	06/24/2021	07/08/2021	09/01/2021	Contracted Covid 19
Vaccine	06/24/2021	07/08/2021	09/02/2021	Contracted Covid 19
Placebo	06/28/2021	07/12/2021	07/26/2021	Contracted Covid 19
Placebo	06/28/2021	07/12/2021	08/13/2021	Received another vaccine
Vaccine	06/28/2021	07/12/2021	08/10/2021	Contracted Covid 19
Vaccine	06/28/2021	07/12/2021	08/04/2021	Received another vaccine
Vaccine	06/28/2021	07/12/2021	08/04/2021	Received another vaccine
Vaccine	06/28/2021	07/12/2021	08/02/2021	Loss to follow-up
Placebo	06/28/2021	07/12/2021	07/12/2021	Contracted Covid 19
Vaccine	06/28/2021	07/12/2021	07/28/2021	Received another vaccine
Placebo	06/28/2021	07/12/2021	08/25/2021	Received another vaccine
Vaccine	06/28/2021	07/12/2021	08/01/2021	Contracted Covid 19
Vaccine	06/28/2021	07/25/2021	08/15/2021	Loss to follow-up
Placebo	06/28/2021	07/12/2021	08/04/2021	Contracted Covid 19
Placebo	06/28/2021	0000/00/00	06/28/2021	Contracted Covid 19
Placebo	06/28/2021	0000/00/00	06/28/2021	Contracted Covid 19

IUM CTC
مرکز کارآزمایی بالینی دانشگاه علوم پزشکی ایران

میلاد دارونور

Placebo	06/28/2021	07/12/2021	08/29/2021	Contracted Covid 19
Vaccine	06/28/2021	07/12/2021	09/13/2021	Received another vaccine
Placebo	06/28/2021	07/12/2021	09/06/2021	Contracted Covid 19
Placebo	06/28/2021	0000/00/00	07/07/2021	Contracted Covid 19
Placebo	07/04/2021	07/18/2021	08/11/2021	Received another vaccine
Vaccine	07/04/2021	07/19/2021	08/09/2021	Loss to follow-up
Placebo	07/04/2021	07/18/2021	08/07/2021	Received another vaccine
Placebo	07/04/2021	07/18/2021	08/23/2021	Received another vaccine
Placebo	07/04/2021	07/18/2021	07/26/2021	Contracted Covid 19
Placebo	07/04/2021	07/18/2021	08/01/2021	Contracted Covid 19
Vaccine	07/04/2021	0000/00/00	07/17/2021	Contracted Covid 19
Vaccine	07/04/2021	0000/00/00	07/12/2021	Contracted Covid 19
Placebo	07/04/2021	0000/00/00	07/04/2021	Contracted Covid 19
Vaccine	07/04/2021	07/18/2021	07/25/2021	Contracted Covid 19
Vaccine	07/04/2021	07/18/2021	08/29/2021	Contracted Covid 19
Vaccine	07/04/2021	07/18/2021	08/04/2021	Received another vaccine
Vaccine	07/04/2021	07/18/2021	08/16/2021	Received another vaccine
Placebo	07/04/2021	07/18/2021	08/08/2021	Loss to follow-up
Vaccine	07/04/2021	07/18/2021	08/14/2021	Contracted Covid 19
Placebo	07/04/2021	0000/00/00	07/10/2021	Contracted Covid 19
Placebo	07/04/2021	07/18/2021	08/02/2021	Received another vaccine
Vaccine	07/04/2021	07/19/2021	08/14/2021	Received another vaccine
Vaccine	07/04/2021	07/18/2021	08/10/2021	Received another vaccine
Vaccine	07/04/2021	0000/00/00	07/04/2021	Contracted Covid 19
Placebo	07/04/2021	07/18/2021	08/12/2021	Received another vaccine
Vaccine	07/04/2021	07/18/2021	08/01/2021	Contracted Covid 19
Placebo	07/04/2021	07/18/2021	08/05/2021	Received Another Vaccine
Placebo	07/04/2021	07/19/2021	08/16/2021	Received Another Vaccine
Vaccine	0000/00/00	0000/00/00	0000/00/00	Loss to follow-up
Placebo	07/04/2021	0000/00/00	07/11/2021	Contracted Covid 19
Placebo	07/04/2021	07/18/2021	08/23/2021	Received Another Vaccine
Placebo	07/04/2021	07/18/2021	08/21/2021	Received Another Vaccine
Vaccine	07/04/2021	07/18/2021	09/01/2021	Contracted Covid 19
Vaccine	07/04/2021	07/18/2021	08/08/2021	Contracted Covid 19
Placebo	07/05/2021	0000/00/00	07/05/2021	Contracted Covid 19
Placebo	07/05/2021	07/19/2021	07/26/2021	Contracted Covid 19
Placebo	07/05/2021	07/19/2021	08/09/2021	Loss to follow-up
Vaccine	07/05/2021	0000/00/00	07/17/2021	Contracted Covid 19
Placebo	07/06/2021	07/20/2021	08/04/2021	Received Another Vaccine
Vaccine	07/06/2021	07/20/2021	08/08/2021	Received Another Vaccine

Vaccine	07/06/2021	07/20/2021	08/11/2021	Contracted Covid 19
Placebo	07/06/2021	07/20/2021	07/27/2021	Contracted Covid 19
Vaccine	07/06/2021	07/20/2021	08/21/2021	Contracted Covid 19
Placebo	07/06/2021	0000/00/00	07/20/2021	Abnormal lab test
Vaccine	07/06/2021	07/24/2021	08/15/2021	Received Another Vaccine
Placebo	07/06/2021	07/20/2021	08/10/2021	Loss to follow-up
Vaccine	07/06/2021	0000/00/00	07/06/2021	Contracted Covid 19
Placebo	07/06/2021	07/26/2021	08/14/2021	Contracted Covid 19
Placebo	07/06/2021	07/26/2021	08/16/2021	Loss to follow-up
Vaccine	07/06/2021	07/20/2021	07/20/2021	Contracted Covid 19
Vaccine	07/06/2021	07/20/2021	08/10/2021	Loss to follow-up
Placebo	07/06/2021	07/20/2021	09/04/2021	Received Another Vaccine
Placebo	07/06/2021	0000/00/00	07/06/2021	Contracted Covid 19
Vaccine	07/06/2021	0000/00/00	07/12/2021	Contracted Covid 19

Survival analysis

Log-rank test

Table S 24 Log rank test comparing the occurrence of symptomatic, PCR-positive Covid-19 two weeks after the second injection in study participants receiving 10 µg/dose vaccine with the placebo group

Study group	Events observed	Events expected	Chi2	P-Value log rank
Vaccine	8	10.95	1.58	0.21
Placebo	14	11.05		

Figure S 22 Kaplan-Mayer survival curve of the occurrence of symptomatic, PCR-positive Covid-19 two weeks after the second injection in study participants receiving 10 µg/dose vaccine compared to the placebo group

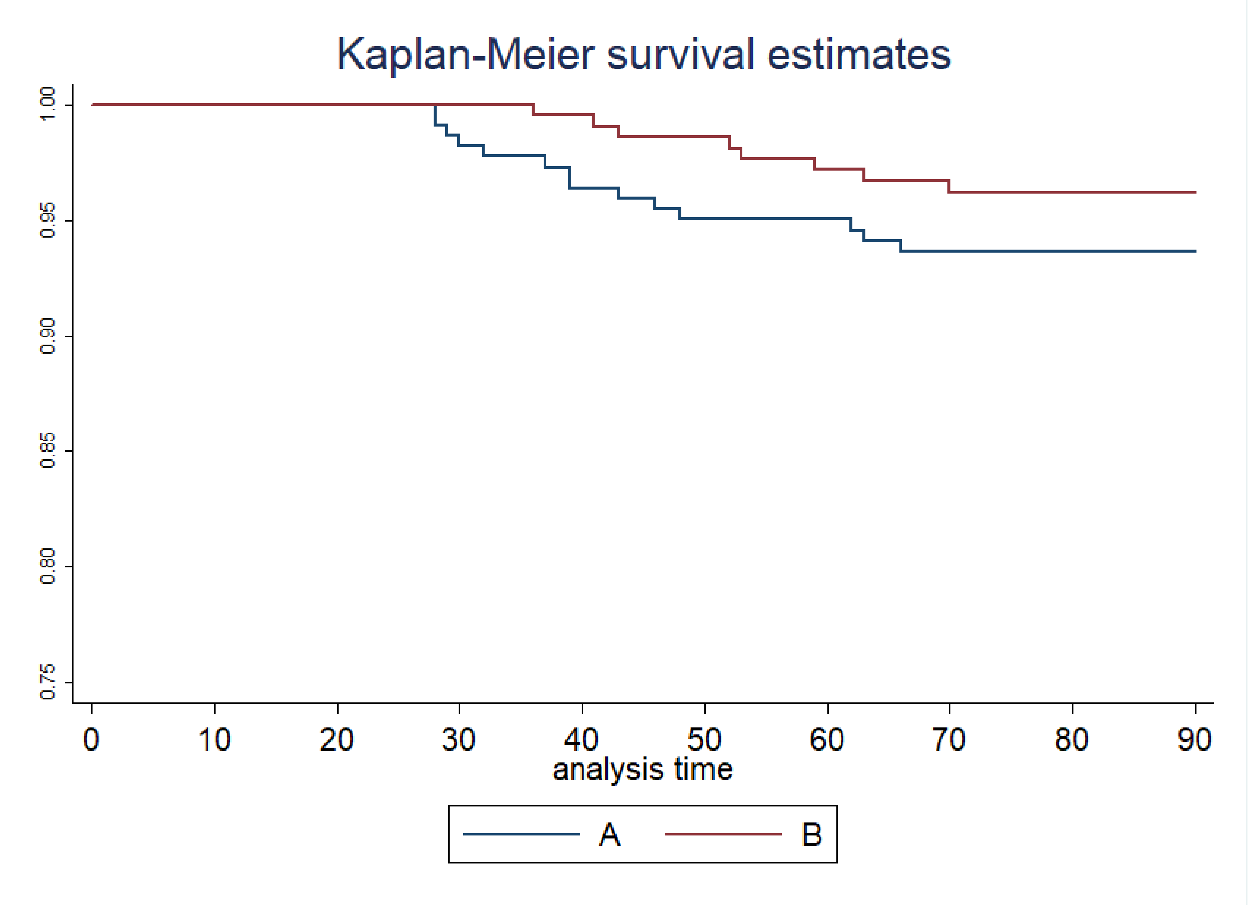

Cox proportional hazard regression

Table S 25 Hazard ratio and its 95% CI estimate derived from cox proportional hazard regression model for the occurrence of symptomatic, PCR-positive Covid-19 two weeks after the second injection in study participants receiving 10 µg/dose vaccine compared to the placebo group

Study group	Haz. Ratio	Std. Err.	Z	P>|z|	[95% Conf. Interval]	
Placebo	1	-	-	-	-	-
Vaccine	0.58	0.26	-1.24	0.21	0.24	1.37
